# Transnasal-brain delivery of nanomedicines for neurodegenerative diseases

**DOI:** 10.3389/fddev.2023.1247162

**Published:** 2023-08-11

**Authors:** Xu Zhang, Maohua Wang, Zhixian Liu, Ying Wang, Li Chen, Jiaqi Guo, Wentao Zhang, Yao Zhang, Chenjie Yu, Tongwu Bie, Youjun Yu, Bing Guan

**Affiliations:** ^1^ Department of Otolaryngology, Head and Neck Surgery, Dalian Medical University, Dalian, China; ^2^ Department of Otolaryngology, Head and Neck Surgery, The First People’s Hospital of Foshan, Hearing and Balance Medical Engineering Technology Center of Guangdong, Foshan, China; ^3^ Department of Otolaryngology, Head and Neck Surgery, Shenzhen Hengsheng Hospital, Shenzhen, China; ^4^ Department of Otolaryngology, Head and Neck Surgery, Clinical Medical College, Yangzhou University, Yangzhou, China; ^5^ Department of Otolaryngology, Head and Neck Surgery, The First Affiliated Hospital of Anhui Medical University, Hefei, China; ^6^ Jiangsu Provincial Key Medical Discipline (Laboratory), Department of Otolaryngology, Head and Neck Surgery, Affiliated Drum Tower Hospital of Nanjing University Medical School, Nanjing, China; ^7^ Department of Otolaryngology, Head and Neck Surgery, Huai’an Second People’s Hospital, The Affiliated Huai’an Hospital of Xuzhou Medical University, Huai’an, China

**Keywords:** nose-to-brain delivery, nanomedicines, exosomes, neurodegenerative diseases, delivery methods, influencing factors, strategies

## Abstract

Neurodegenerative diseases (NDs) have become a serious global health problem as the population ages. Traditionally, treatment strategies for NDs have included oral and intravenous administration; however, the blood–brain barrier (BBB) can prevent drugs from reaching the brain, rendering the treatment incomplete and the effect unsatisfactory. Additionally, the prolonged or excessive use of drugs that can cross the BBB can damage liver and kidney function. Recent studies have shown that nose-to-brain drug delivery can noninvasively bypass the BBB, allowing drugs to enter the brain through the olfactory or trigeminal nerve pathways; additionally, nanoparticle carriers can enhance drug delivery. This review introduces drug carrier nanoparticles for nose-to-brain delivery systems, compares the advantages and disadvantages of different nanoparticles, and discusses the factors influencing nose-to-brain nanomedicine delivery and enhancement strategies. We also summarize nose-to-brain delivery and nanomedicines for treating NDs, the current challenges of this approach, and the future promise of nanomedicine-based ND treatment.

## 1 Introduction

Alzheimer’s disease (AD), Parkinson’s disease (PD), Huntington’s disease (HD), and sensorineural hearing loss (SNHL) are neurodegenerative diseases (NDs) caused by the accumulation of misfolded proteins inside and outside cells, and by the deformation or loss of neurons (Teleanu et al., 2022). As the global population ages, the prevalence of neurodegenerative diseases is expected to rise (Noble and Burns, 2010). According to the World Health Organization, 57.4 million people worldwide were living with neurodegenerative diseases in 2019 and this number is expected to rise to 153 million by 2050 (A et al., 2022). The corresponding healthcare costs have increased with the increasing prevalence of NDs, significantly impacting patients, families, and society.

NDs cause neurological and psychiatric symptoms depending on the area of the brain affected, mainly progressive motor dysfunction and cognitive impairment. Pharmacological agents are the main means of treating these disorders, traditionally via oral and intravenous administration; however, therapeutic efficacy is hampered by the blood–brain barrier (BBB). The BBB is a unique microvascular system in the brain that regulates the movement of ions, molecules, and cells between the blood and the brain (Profaci et al., 2020). Although the BBB protects the brain from toxins and pathogens, it also poses a serious obstacle to delivering therapeutic drugs, with 98% of small molecules and almost all large molecules unable to cross the BBB. Other factors to be considered during the delivery of drugs to the brain via the circulatory pathway include minimal peripheral exposure, first-pass metabolism, plasma protein binding, and rapid elimination (Tan et al., 2020).

Numerous methods for delivering drugs to the brain have been reported recently. In contrast to conventional drug delivery via the circulatory pathways, intranasal drug delivery bypasses the BBB and targets the brain directly (Perteghella et al., 2021). Since the drug does not reach non-targeted sites, the systemic side effects and dose are reduced. Better bioavailability of the drug is obtained through intranasal administration, as it avoids first-pass metabolism and overcomes incomplete absorption in the gastrointestinal tract (Homayun et al., 2019). This noninvasive approach facilitates self-administration, especially for patients with motor disorders, nausea, impaired gastrointestinal function, and salivary gland dysfunction (Hoekman et al., 2020). Intranasal delivery results in a higher drug concentration than oral administration at the same dose (Bicker et al., 2020).

Nanoparticles are emerging drug delivery vehicles that resolve various obstacles in the drug delivery process. Nanoparticles can protect the delivered drug from degradation by biological or chemical factors in the nasal environment and from the effects of efflux proteins and release and modulate the drug faster, which is advantageous in emergencies and long-term dosing situations. In addition, nanoparticles can facilitate targeted drug transport, thus increasing the efficiency and controllability of nose-to-brain delivery (Bicker et al., 2020).

There are few studies on the nose-to-brain delivery of nanomedicines to the brain for treating NDs. Most current studies are animal experiments; thus, these approaches are far from translation into clinical applications. While nose-to-brain delivery has advantages and limitations, few studies have investigated the limitations and how to overcome them.

Therefore, here, we review the different nose-to-brain delivery pathways and compare the advantages and disadvantages of different nanocarriers. In addition, we summarize the strategies and methods for nose-to-brain delivery for treating NDs and discuss the challenges and potential directions for intranasal nanomedicine delivery.

## 2 Route of nose-to-brain delivery

### 2.1 Anatomy of the nasal cavity

The nasal epithelium can be divided into three regions based on the cell types present: the nasal vestibule, the respiratory epithelium, and the olfactory epithelium (Lofts et al., 2022). The nasal vestibule is the most anterior part of the nasal cavity. The small surface area and low vascularization of the nasal vestibule make drug absorption negligible. The respiratory epithelium is located in the posterior part of the nasal vestibule. Since the respiratory epithelium has a surface area of approximately 130 cm^2^, accounts for 90% of the nasal area, and is extensively vascularized, it is considered the primary site for drug delivery. The ophthalmic and maxillary nerves, which are branches of the trigeminal nerve, innervate this region; thus, drugs can be delivered to the brain via the trigeminal pathway. The olfactory epithelium is located in the upper posterior part of the nasal cavity, with an area of approximately 10 cm^2^, accounting for 10% of the entire nasal epithelium. Olfactory sensory neurons (OSNs) are responsible for the perception and transmission of odor information to the brain via the olfactory pathway, which comprises OSNs, the lamina propria, and the olfactory bulb that projects to various brain regions. This pathway is a direct route for drug delivery to the brain. Since the lamina propria of the olfactory epithelium also houses the maxillary nerve, therapeutic drugs can be delivered to the brain via the trigeminal pathway.

### 2.2 Drug delivery pathway through the nasal cavity to the brain

**FIGURE 1:**
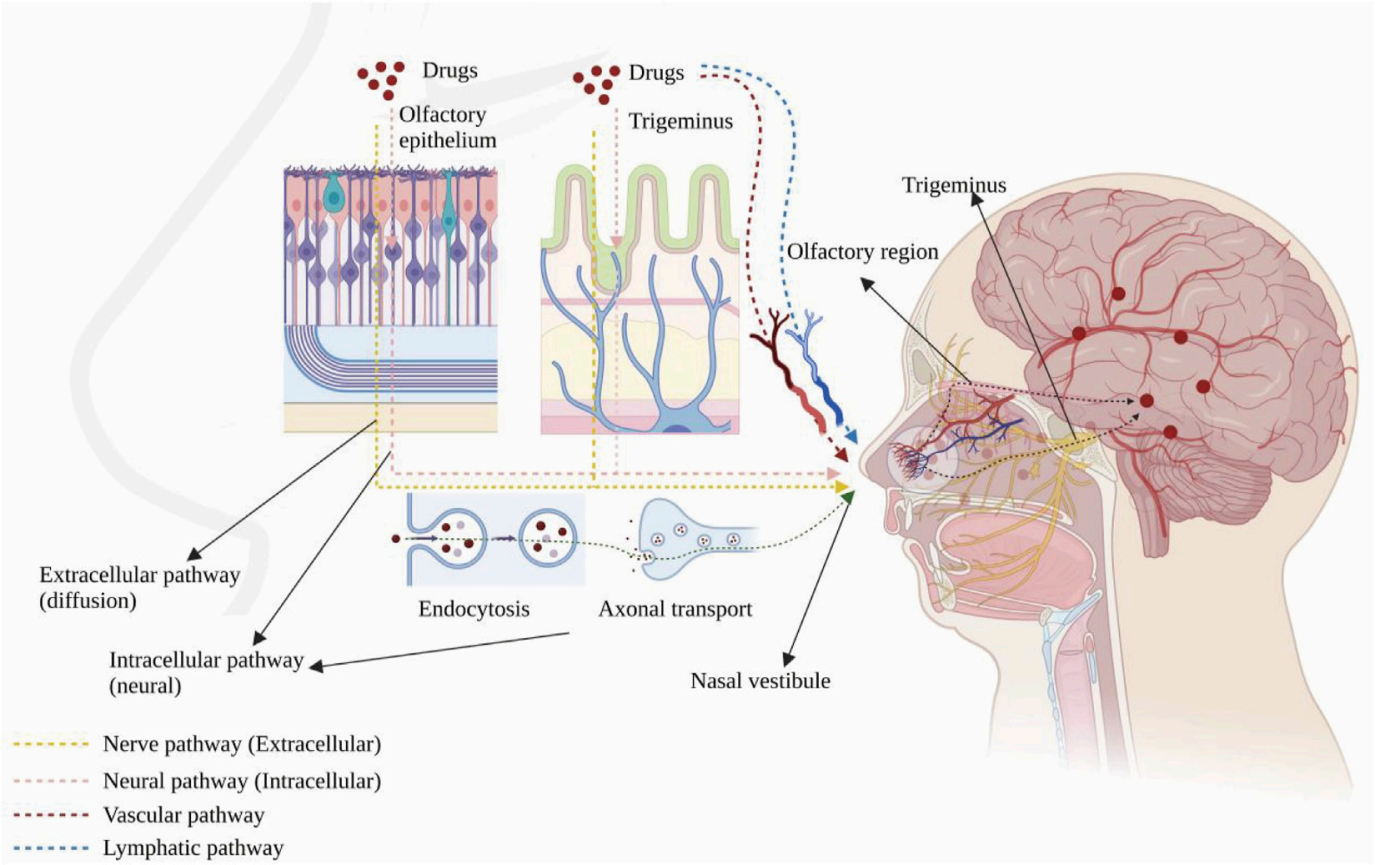
Anatomy of the human nasal cavity and four nose-to-brain delivery pathways. Created with BioRender.com.

Drugs can reach the brain via indirect and direct pathways after reaching the sieve plate via the nasal mucosa (Bicker et al., 2020). In the indirect pathway, some drugs are absorbed into the vascular or lymphatic systems, enter the systemic circulatory system and reach the brain through the BBB. In the direct pathway, drugs bypass the BBB through the nasal mucosa and are directly connected to the nerves in the brain and spinal cord and delivered through the neural pathways (olfactory and trigeminal). After drugs are delivered to the brain, they disperse via intracellular or extracellular delivery mechanisms. Intracellular delivery refers to the entry and exit of drugs into and out of neurons by cytokinesis or receptor-mediated transport, mainly through axons, which leads to the central nervous system (CNS). The drug is then further dispersed by fluid movement (Kashyap and Shukla, 2019). Extracellular delivery involves the drug crossing the nasal epithelium to the lamina propria, where neurons are located. Then, the drug is transported along neuronal axons through the overall flow transport process via perineural channels, through which the drug can be transported from one neuron to another. Drug delivery via the nasal cavity involves olfactory and trigeminal nerve pathways, vascular pathways, cerebrospinal fluid, and lymphatics (Figure 1).

#### 2.2.1 Olfactory nerve pathway

Drugs are transported across the sieve plate via the olfactory nerve to the olfactory bulb in the brain (Lofts et al., 2022). The drug concentration in the olfactory epithelium positively correlates with its concentration in the olfactory bulb (Cunha et al., 2017). A study indicated that DL-3-n-butylphthalide applied to the nasal mucosa are able to enter the brain regions through the olfactory nerve pathway, olfactory epithelium pathway and bloodstream, making it possible for central nervous diseases treatment (Zhao et al., 2017). Drugs can also be ingested by cytokinesis or passive diffusion and axonally transported to the olfactory bulb and other brain regions. Metal salts, such as aluminium salt (Das et al., 2021), and those with the relevant receptor (wheat germ agglutinin conjugated to horseradish peroxidase, WGA–HRP) are delivered in this way (Shu et al., 2019).

#### 2.2.2 Trigeminal nerve pathway

The CNS innervates the respiratory and olfactory epithelium through the trigeminal nerve (Patel, 2017). Drugs can be transported to the brain via these two sites, and the delivery mechanism is similar to that of the olfactory neural pathway. IGF-I was the first drug to be delivered intranasally via the trigeminal nerve pathway (Thorne et al., 2004) and was observed in the trigeminal branches and ganglia. In addition, another study also found a high concentration of the drug in the caudal medulla after intranasal administration, demonstrating that drugs can be delivered via the trigeminal pathway after intranasal administration (Charlton et al., 2008).

#### 2.2.3 Vascular pathways

Blood from the branches of the maxillary, facial, and ophthalmic arteries and the carotid artery supplies the nasal cavity (Patel, 2017), and drugs delivered through this pathway mainly act on the respiratory epithelial area, which has a large surface area and abundant blood vessels (Lofts et al., 2022). As with intravenous administration, after the drug enters the circulation, it is partially metabolized by the liver and kidneys, degraded by plasma proteases, and bound by plasma proteins; some absorbed drugs do not cross the BBB, reducing the dose that reaches the CNS. Small lipophilic drugs are more likely to cross the BBB than large molecules, such as peptides and proteins (Patel, 2017).

#### 2.2.4 Pathways involving cerebrospinal fluid and lymphatics

The nasal lymphatics are connected to the cerebrospinal fluid, and there are perineurial spaces in the olfactory nerve through which drugs can enter the CNS directly (Wang et al., 2023). A study has found that intranasal administration can promote the absorption of drugs in Nao-Qing microemulsion and achieve fast effect (Li et al., 2015).

### 2.3 Safety of nose-to-brain administration

#### 2.3.1 Toxicity

Most NDs require long-term treatment; thus, toxicity is a concern, including the toxicity of the drug and excipients related to the formulation, such as preservatives, surfactants, and mucoadhesives. Repeated delivery of the drug to the nasal cavity can cause nasal pruritus, nasal bleeding, reduced or altered sense of smell and taste, sinusitis, and nasal epithelial damage (Musumeci et al., 2019). Some drugs also alter ciliary beating frequency (e.g., atropine) (Ukai et al., 1985) and inhibit ciliary motility (e.g., ketamine) (Feldman et al., 2021), and enhancing long-term patient compliance is a challenge that requires further evaluation.

#### 2.3.2 Inappropriate drug use by patients

Due to the small surface area of the olfactory epithelium and the large area of the respiratory epithelium, the inappropriate administration of drugs that act on the neural pathway can lead to absorption in the highly vascularized respiratory area, reducing the absorption of drugs through the nasal pathways and increasing the circulatory side effects (Lofts et al., 2022). The development of appropriate drug delivery devices and patient education is necessary.

#### 2.3.3 Airway hazards

Air enters the airway via nasal breathing in most mammals, including humans. Therefore, treatment of NDs via nose-to-brain administration can interfere with the patient’s normal breathing pattern. For adults, nasal resistance accounts for half of the airway resistance; thus, even a slight change in nasal resistance from the drug can cause dramatic changes in breathing. In addition, due to drug toxicity, repeated use can cause rhinitis, which endangers the patient’s breathing safety. Moreover, nasal circulation can help hydrate the airway surface, and the two nasal cavities can take turns assuming air conditioning and mucus removal functions, jeopardizing the patient’s airway safety if the two nasal cavities are administered unequal doses (White et al., 2015). Therefore, developing individualized protocols to minimize airway hazards is extremely important.

## 3 Treatment for central nervous system disease

### 3.1 Nanocarriers for nose-to-brain drug delivery

**FIGURE 2:**
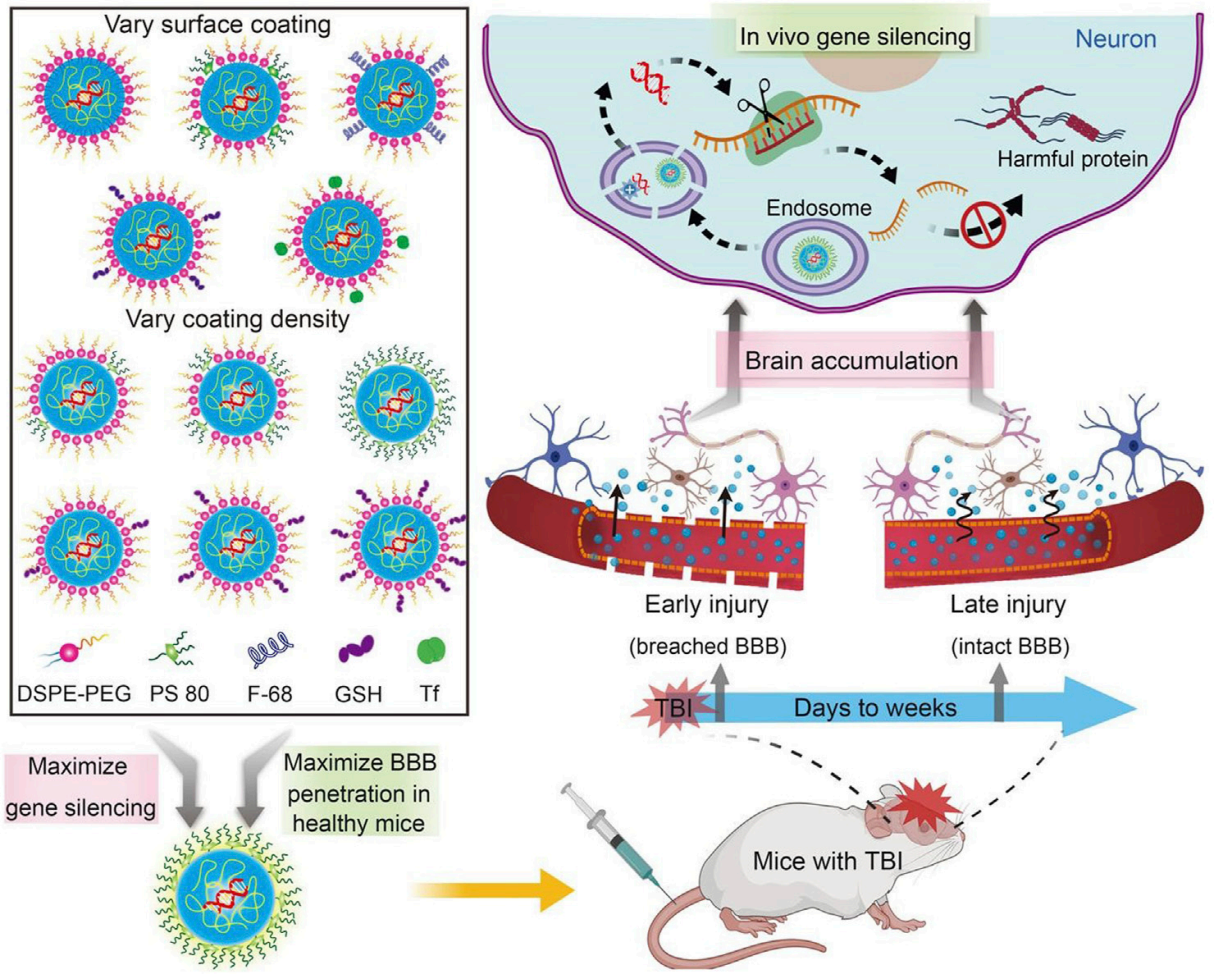
Nanocarriers for drug delivery to the brain across the blood-brain barrier. Reproduced with permission (Wen et al., 2021). Copyright 2021, Science advances.

**FIGURE 3:**
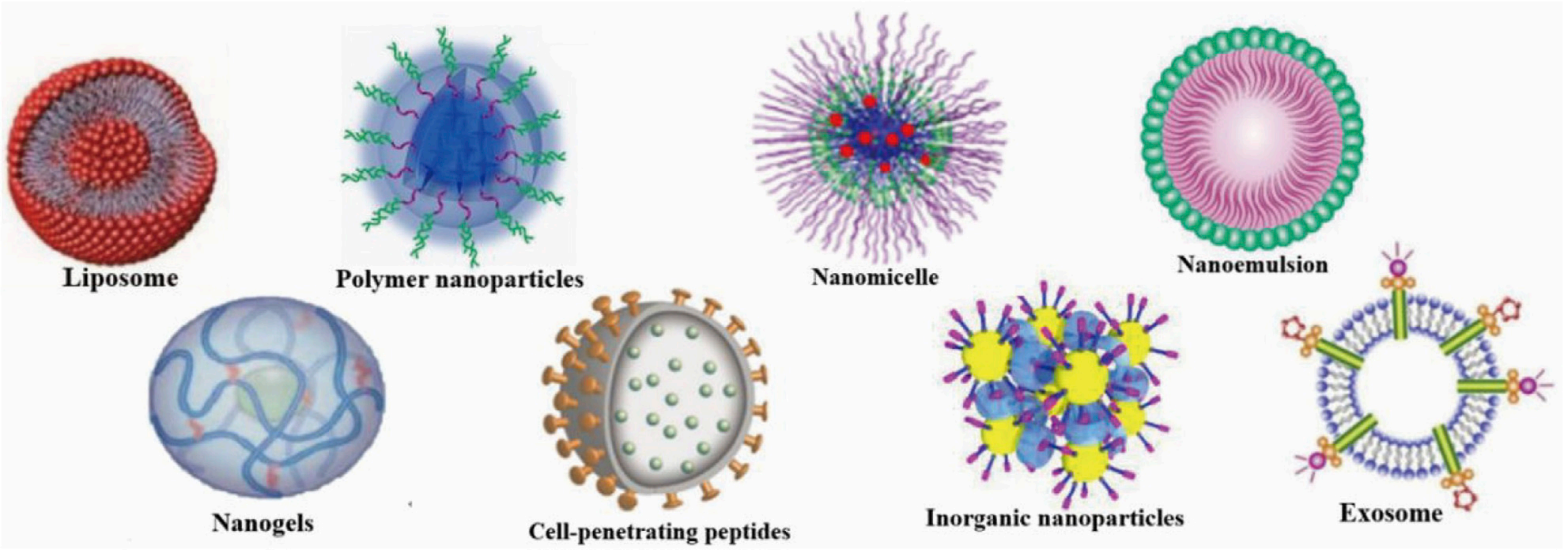
Nanocarriers for nose-to-brain drug delivery. Created with BioRender.com.

Drug delivery systems deliver a certain dose to a target site in the body and maintain the drug concentration in that area at a dose appropriate for the duration of the treatment (Moritz and Geszke-Moritz, 2022). Nanocarriers are loaded with drugs to achieve this purpose and to deliver drugs to the brain efficiently, where they are selectively released to the target site via transport to specific cells and tissues, avoiding side effects caused by systemic exposure; nanocarriers also help drugs cross the BBB and provide neuroprotection (Figure 2) (Wen et al., 2021). Therefore, various nanocarriers have been investigated for nose-to-brain drug delivery systems (Riccardi et al., 2021; Correia et al., 2022), including organic or inorganic nanostructures. Polymeric nanoparticles, lipid nanoparticles, micelles, nanoemulsions, nanogels, cell-penetrating peptides and exosomes belong to organic nanostructures. Inorganic nanoparticles consist of inorganic matter (Figure 3). Here, we summarize these nanocarriers and compare their advantages and disadvantages (Table 1).

**TABLE 1:** Comparison of the advantages and disadvantages of nanocarriers for nose-to-brain drug delivery.

Nanocarriers	Properties	Advantages	Disadvantages
Polymeric nanoparticles	Polymer	1. Combination of various, more stable than single-molecule carriers	1. Instant drug release, not sustained drug release
		2. Can deliver multiple substances with high loading capacity	2. High cost, difficult to produce on a large scale
		3. Ligand-modified, targetable, anti-degradation, low-toxic	
Lipid nanoparticles	For delivery of lipophilic drugs	1. Wide range of raw materials, easy to produce	1. Unstable, easily degraded
		2. High biocompatibility and safety	2. Difficult to package water-soluble substances
		3. Controlled drug release for a long time	3. Weak ability to release drugs instantly
		4. Large surface area and high ability to penetrate the BBB	
Micelles	Assembled from lipid nanoparticles and core surfactant	Smaller than other nanoparticles	1. Less biocompatible than lipid nanoparticles
			2. Potential toxicity of the core surfactant
Nanoemulsions	Liquid lipid	1. Can be made into a spray for easy drug delivery	Requiring a large amount of surfactant to prepare makes the drug ineffective when passing through biofilm
		2. Mucoadhesion to increase drug retention time in the nasal cavity to improve drug bioavailability	
Nanogel	Crosslinked polymer network	1. High hydration, high deformation ability, high loading capacity to deliver multiple drugs	1. Unstable integration, acid-sensitive and reductive, easily degraded
		2. High ability to penetrate the BBB and continuous drug release	2. Component materials can cause some damage to the nasal cavity
Cell-penetrating peptide	Compounded with selected drugs	1. Penetrating cell membranes with high biocompatibility and safety	1. Unstable
		2. High ability to penetrate BBB	2. Can enter all cells with toxicity and lower drug concentration at the specified site
		3. targetable	
Inorganic nanoparticles	Inorganic material	1. Specified material composition, easy to produce, more stable compared to organic materials	1. Difficult to remove and toxic
		2. Mesoporous inorganic nanoparticles can be targeted, high loading capacity and biocompatibility, easy diffusion in the brain	2. Uncontrolled drug release
Exosomes	A single lipid membrane vesicle secreted by cells	1. Can be fused with cells with high biocompatibility and safety	Difficulty in targeting delivery
		2. Readily crosses the BBB	
		3. Can be used in multiple ways to treat NDs with high potential	

#### 3.1.1 Polymer-based nanocarriers

Polymer-based nanocarriers can be optimally combined with drugs and are very stable (Patel et al., 2012). They can be loaded with larger drug levels than single-molecule carriers. In addition to delivering drugs, polymers can potentially deliver nucleic acids, proteins, and diagnostic reagents (Fahmy et al., 2007; Demento et al., 2009; Woodrow et al., 2009). Several researchers utilized Polylactic-co-glycolic acid (PLGA) nanoparticles to load with siRNA and lead to efficient and sustained gene silencing (Woodrow et al., 2009). Additionally, Polymer-based nanocarriers can be modified with ligands, and many are safe for long-term use in humans (Patel et al., 2012). For example, modified chitosan is often used to treat neurological diseases (Manek et al., 2020). Polymer-based nanocarriers modified with nanoparticles can protect the drug from chemical or enzymatic degradation, making it easier for the active molecule to reach the target site, thus increasing therapeutic efficacy (Tosi et al., 2008). Polymer-based nanocarriers modified with nanoparticles can facilitate drug targeting and enhance therapeutic effects (Tosi et al., 2008). Treating polymer surfaces with appropriate ligands can reduce toxicity and avoid rapid clearance *in vivo* (Zhou et al., 2018). Polyalkylcyanoacrylate (PACA) is one of the most common NP modifications for delivery to the CNS (Vauthier et al., 2007). PLGA and polylactic acid (PLA) are the most commonly used polymers (Mirhadi et al., 2018). PLA and PLGA have been approved for clinical use in humans; their degradation products are readily removed in the body, and they do not produce inflammatory reactions (Dechy-Cabaret et al., 2004). Chitosan nanoparticles can easily cross the BBB due to their large positive charge (Yu et al., 2019). However, polymers often exhibit instantaneous rather than sustained drug release (Correia et al., 2022). In addition, the high cost and mass production difficulties associated with some polymers make them difficult to apply in the market (Fernandes et al., 2021).

#### 3.1.2 Lipid nanoparticles

Lipid nanoparticles are nanocarriers that can deliver and store different lipophilic drugs (Briuglia et al., 2015). The materials used for their fabrication are simple and widely available; thus, these nanoparticles are easy to produce industrially (Fernandes et al., 2021; Correia et al., 2022). They can be prepared without organic solvents, are easily degraded and removed, and have better biocompatibility, exhibiting a higher safety profile than polymeric nanoparticles. The ability of lipid nanoparticles to encapsulate drugs is superior to other approaches and allows controlled drug release over a longer period (Naseri et al., 2015). However, lipid nanoparticles are unstable and susceptible to degradation by environmental factors, including unsuitable storage temperatures and pH and exposure to light or oxygen (Liu et al., 2015). Additionally, it is difficult to package highly water-soluble substances and achieve instantaneous drug release with lipid nanoparticles (Campardelli et al., 2016).

#### 3.1.3 Micelles

Nanomicelles, comprising polar and nonpolar molecules, are erelatively dense spherulitic structure, and drug were entrapped within nanomicelles (Lofts et al., 2022). The surfactant determines micelle size; generally, micelles are smaller than other nanocarriers. However, regarding human drug delivery, micelle cytocompatibility is low, and the dose of the delivered drug and potential surfactant toxicity determines the ease of fabrication. Poly (ethylene glycol)-block-poly (D, L-lactide) (PEG–PLA) micelles can deliver baicalein to the brain via the nose for the treatment of NDs caused by oxidative stress and inflammation (Zhang et al., 2020). In addition, hydroxytyrosol micelles protect against Parkinson-related oxidative stress *in vitro* (Mursaleen et al., 2021).

#### 3.1.4 Nanoemulsions

Nanoemulsions are drug carriers made using liquid lipids with a similar structure to lipid nanoparticles (Bonferoni et al., 2019) that can be made into a spray for easy intranasal administration (Makidon et al., 2010). Nanoemulsions can also incorporate components that adhere to the nasal mucosa (Bahadur et al., 2020), leading to increased bioavailability in nanoemulsion-mediated intranasal drug delivery, prolonging the retention time and facilitating nose-to-brain drug delivery in the olfactory and trigeminal nerve pathways (Bonferoni et al., 2019). Some nanoemulsions can also be absorbed directly into the lymphatic system, avoiding first-pass metabolism to improve bioavailability (Singh et al., 2017). Several nanoemulsions are beneficial in neurodegeneration, including nimodipine, curcumin, resveratrol, selegiline, and rivastigmine (Pangeni et al., 2014; Pathak et al., 2014; Kumar et al., 2016; Bonferoni et al., 2019). However, there are obvious drawbacks to using nanoemulsions in humans, such as lower biocompatibility than lipid nanoparticles and the potential toxicity of core surfactants; thus, it must be ensured that the product is nontoxic (Singh et al., 2017). The preparation of nanoemulsions usually requires a large volume of surfactant to stabilize the droplets; however, when passing through a biofilm, the surfactant can fluidize, making the emulsion ineffective for use in the human body.

#### 3.1.5 Nanogels

Nanogels are crosslinked polymer networks with high hydration, deformability, and loading capacity and can release drugs over a longer period, thus prolonging the treatment (Lofts et al., 2022). Nanogels have been widely used to deliver various drugs and diagnostic reagents (Neamtu et al., 2017) and release oligonucleotides, proteins, low molecular weight drugs, and other small molecules after dissolution in water (Song et al., 2015; Neamtu et al., 2017). Nanogel products are nontoxic in living organisms. Poly (N-vinylpyrrolidone)-based (Picone et al., 2018) and carboxylated poly (N-vinyl pyrrolidone) (Picone et al., 2016) nanogels have been used to treat NDs; these nanogels transport drugs from the nose to the brain via olfactory and trigeminal nerve pathways. However, unstable bonds were incorporated into the crosslinked polymer network to make the nanogels degradable, making the inherently reductive nanogels sensitive to acidic conditions (Tahara and Akiyoshi, 2015; Neamtu et al., 2017) and susceptible to degradation during nose-to-brain drug delivery. In addition, the safety of the excipients of nanogels for intranasal administration to nasal epithelial cells is unclear (Aderibigbe, 2018).

#### 3.1.6 Cell-penetrating peptides

Cell-penetrating peptides (CPPs) can form complexes with selected drugs for intranasal drug delivery (Sharma et al., 2016), penetrate cell membranes, and transfer into cells, thus increasing drug biocompatibility. CPPs can help drugs to cross physiological barriers, such as the BBB and nasal mucosa (Zhang et al., 2016). CPPs can also be combined with nanomedicines to increase their targeting, enhancing delivery efficiency (Douat et al., 2015). Cyclic adenosine monophosphate (CAMP), PEP-1-Paraoxonase 1 (PEP-1-PON1), K16 Apolipoprotein E (K16ApoE), transactivator of transcription (TAT), and other CPPs can effectively improve drug delivery for treating NDs (Xie et al., 2020). However, CPPs are prone to internal degradation when exposed to the blood because their peptide nature is unstable (Kristensen et al., 2016); thus, drug degradation via the indirect pathway is less efficient as an insufficient drug concentration is targeted to the localization site. In addition, CPPs can enter almost all cells in the body, which is harmful. Similarly, CPPs are widely distributed in many cellular tissues, reducing the drug concentration at the indicated site and reducing efficiency.

#### 3.1.7 Exosomes

Exosomes are special drug carriers comprising cell-secreted single lipid membrane vesicles that can easily move from 1 cell to another (Lässer et al., 2011). The fusion process observed in R18-labeled exosomes and PKH-67-labeled cells revealed that exosomes bind to target cells via membrane receptors and fuse directly with the plasma membrane or release their contents by endocytosis, thus resulting in very high biocompatibility (Parolini et al., 2009). Adding curcumin to exosomes increases its biocompatibility, thereby increasing the efficiency of drug delivery to the brain (Sun et al., 2010). Exosomes can also enhance the drug retention time in the nasal mucosa, increasing bioavailability (Zhuang et al., 2011). Exosomes can cross the BBB to deliver nucleic acids, such as miRNA and siRNA. Modified exosomes have been used to deliver exogenous siRNA to brain tissue in AD mice via the intravenous route (Alvarez-Erviti et al., 2011). Exosomes are also widely used to treat NDs, and exosomes from N2a cells or human cerebrospinal fluid can reduce the accumulation of intracellular β-amyloid (Aβ) through surface proteins, such as cellular prion protein (PrPC), which can play a role in AD treatment (An et al., 2013). Exosomes can deliver gamma interferon (IFN-γ) stimulated dendritic cells to the CNS, improving myelin regeneration (Pusic et al., 2021). Sphingolipid metabolizing enzymes can modulate exosomes to direct conformational changes in Aβ and promote the uptake of Aβ by microglia to reduce Aβ levels in the AD brain (Yuyama et al., 2012). Exosomes isolated from adipose-derived stem cells (ADSCs) contain high levels of neutral endopeptidase (NEP) (Katsuda et al., 2013), which eliminates Aβ (Iwata et al., 2001), and rat neural stem cells have high levels of cystatin C, which can nourish the brain and nerves (Taupin et al., 2000). Exosomes delivered via the nose encapsulate synaptic proteins and are released in response to high neuronal activity or stimulation by oxidative stress, promoting neuronal growth and neuronal survival and keeping neurons free from oxidative stress (Wang et al., 2011). In addition, exosomes loaded with catalase delivered to the brain have neuroprotective effects and may be effective for treating NDs (Haney et al., 2015). Intranasal delivery of human-induced pluripotent stem cell–derived exosomes to the brain has been shown to yield better bioavailability than the intravenous route in mice (Gu et al., 2022), and this approach facilitated easier clinical acceptance. In conclusion, exosomes are a promising drug carrier; however, achieving high selectivity for target cells is challenging (Jiang L. et al., 2019).

#### 3.1.8 Inorganic nanoparticles

Inorganic nanoparticles are composed of different inorganic materials, such as silica and metal oxides, that are uniform in size, easy to produce, and more stable in structure than organic materials (Kim et al., 2019). Among them, mesoporous silica nanoparticles, which are inorganic nanoparticles used to deliver drugs, can provide greater holding space, better biocompatibility, and easier-to-impose surface functionalization for brain-targeted transport (Alexander et al., 2019). These nanoparticles can diffuse better than others in the brain, facilitating drug distribution (Fahmy et al., 2019). However, inorganic nanoparticles are toxic to humans, drug release is difficult to control, and complete clearance from the body is difficult (Yang et al., 2010).

### 3.2 Methods for nose-to-brain delivery

It is necessary to increase the exposure time between the drug and the nasal mucosa and to target the drug effectively to specific regions via the nose to maximize the therapeutic efficacy of nose-to-brain drug delivery.

#### 3.2.1 Promoting mucoadhesion

Mucoadhesion can be divided into two steps. First, the two surfaces are in close contact, and consolidation results in firm binding (Smart, 2005). The prolonged retention time of nanocarriers at the absorption site typically increases the bioavailability of the carried drug. Therefore, the mucoadhesion of nanomedicines may facilitate nasal absorption and increase the retention time of the drug in the brain, and the frequency of drug administration by the patient can be reduced.

**FIGURE 4:**
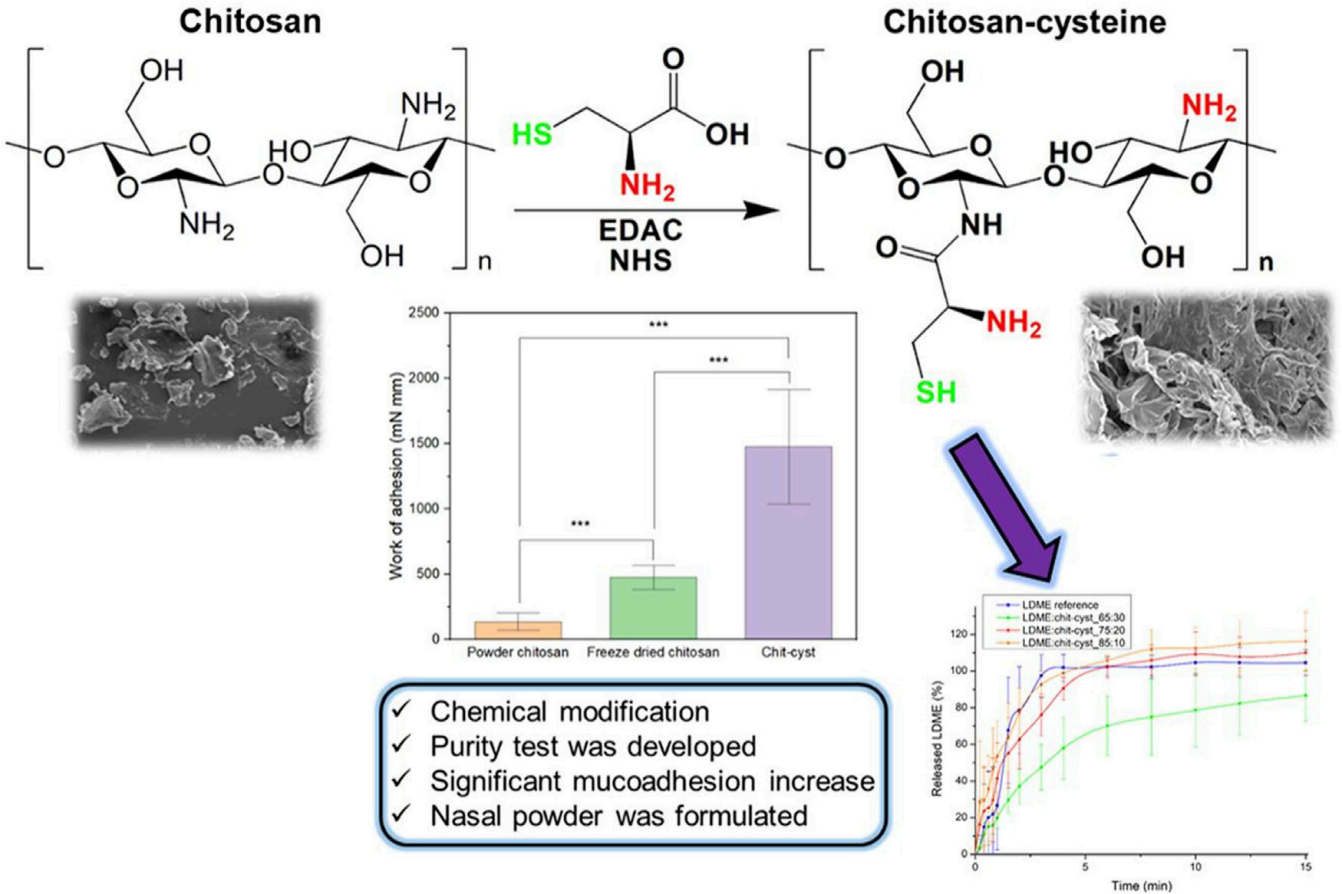
Thiol chitosan-cysteine as a suitable mucoadhesion excipient for nasal dry powder. Reproduced with permission (Kiss et al., 2022). Copyright 2022, Elsevier.

In addition to the adherence of drugs to mucosal surfaces conferred by different nanocarriers, nasal microspheres can be combined with nanocarriers to achieve better mucoadhesion (Gangane et al., 2020). Nasal microspheres enter the nasal cavity and contain cations that bind to the nasal secretion and undergo spontaneous gelation in the nasal cavity, prolonging residence time. Polymers combined with microspheres can obtain better results, and hydroxypropyl beta-cyclodextrin (HP-CD) polymer microspheres of chitosan or sodium alginate inhibit hippocampal oxidative stress and apoptosis and counteract Ab-induced neurotoxic effects in AD (Yalcin et al., 2016). Lectin-functionalized microspheres enhance retention and show a twofold efficiency increase in nasal mucoadhesion compared with non-functionalized microspheres (Gao et al., 2021). Nasal powders also achieve mucoadhesion (Figure 4) (Kiss et al., 2022). Recent studies have shown that chitosan–cysteine adducts made with the anti-Parkinson’s disease drug levodopa methyl ester hydrochloride increase mucoadhesion by forming disulfide bonds and show better adhesion effects than deacetylated chitosan powders (Kiss et al., 2022). However, this approach has not been studied in animal models.

#### 3.2.2 Stimulus-responsive drug carriers

**FIGURE 5:**
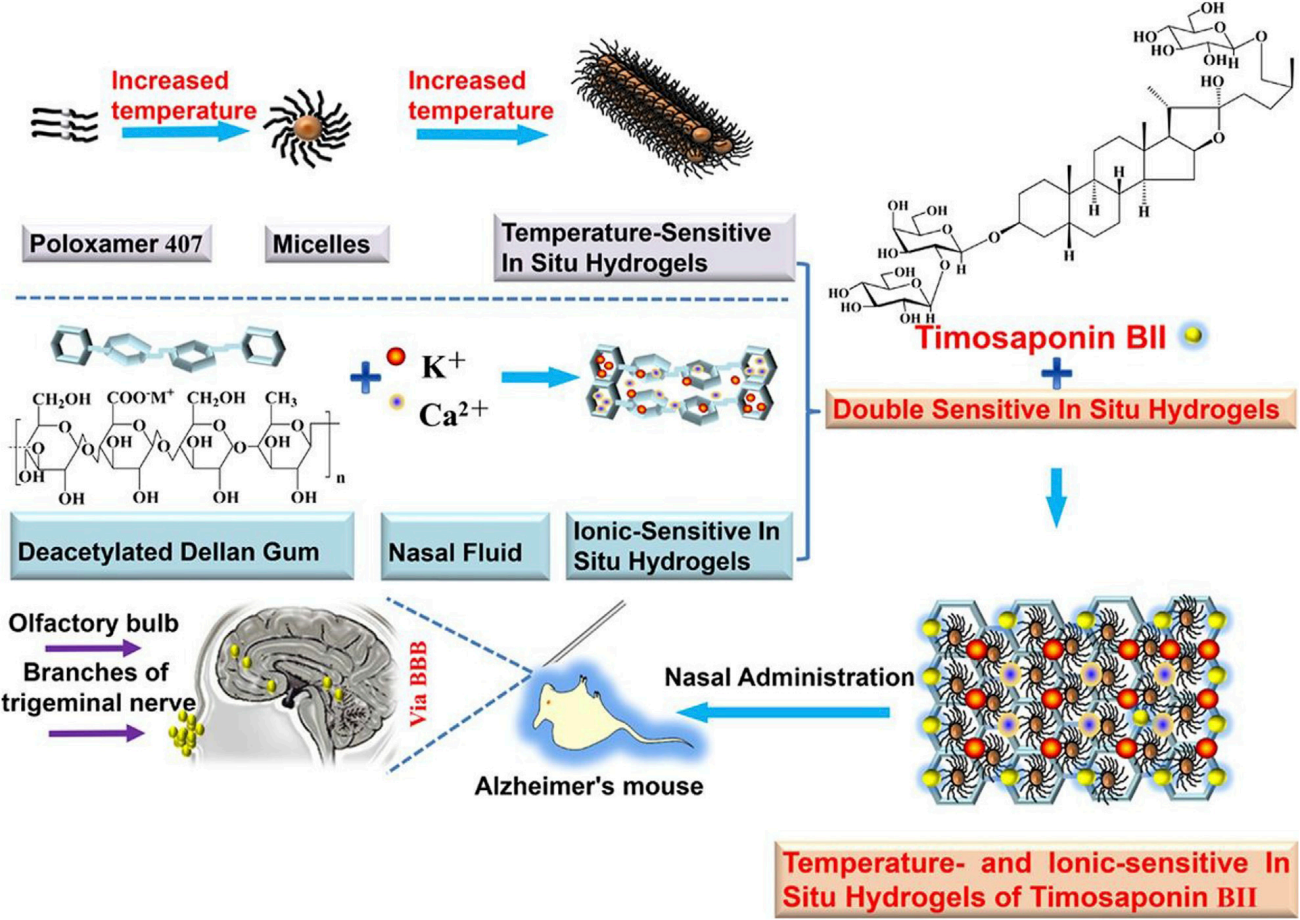
Temperature/ion dual-sensitive *in situ* hydrogel of nasal timosaponin BII. Reproduced with permission (Chen et al., 2020). Copyright 2020, Elsevier.

Stimulus-responsive carriers are a class of drug delivery systems that change physicochemical properties in response to a specific stimulus (Sheng et al., 2019). These changed properties can be adapted to the nasal environment to facilitate nose-to-brain drug delivery. *In situ* hydrogels have been formulated with the anti-AD drug nasal timosaponin BII via temperature or ionic stimulation (Figure 5) (Chen et al., 2020); the temperature and ionic environment in the nasal cavity stabilized the drug, significantly increasing the retention time. An *in situ* gel loaded with the anti-AD drug tacrine prepared by the thermosensitive polymer Pluronic F-127 increased the retention time in the nasal cavity and absorption in the brain (Qian et al., 2014). A composite gel loaded with the anti-AD drug rivastigmine tartrate facilitated absorption better than regular gel (Salatin et al., 2017). *In situ* preparation of a thermoreversible nasal gel adhered to the nasal mucosa carrying Parkinson’s disease drugs increases the adhesion effect and protects the nasal mucosa more than in normal nasal drug delivery (Rao et al., 2017). A thermosensitive gel used in lecithin–chitosan hybrid nanoparticles loaded with pyridoxine for treating PD improved the therapeutic effect of nasal brain delivery (Uppuluri et al., 2021). In addition, cubic phase *in situ* nasal gels based on mucoadhesion are expected to be new vehicles for nose-to-brain drug delivery for NDs (Patil et al., 2019). However, a study of levodopa-configured thermoreversible gels for PD showed that highly viscous gels are detrimental to drug absorption in the nasal cavity (Sharma et al., 2014). Therefore, gel safety needs to be further investigated.

#### 3.2.3 Targeted ligand functionalized nanocarriers

Modifying the nanocarrier surface with targeting ligands after covalent modification can optimize the delivery of nanocarriers. The first targeting ligand used as a nanocarrier was wheat germ agglutinin (WGA), mainly on the surface of the olfactory epithelium (Schwab et al., 1978). One study revealed that after intranasal administration, WGA-modified nanoparticles, compared to unmodified nanoparticles, were more abundant in the brain (Gao et al., 2007). Furthermore, tracking the location of these nanocarriers in the brain revealed particularly high accumulation at the olfactory bulb, suggesting that they mainly target the olfactory pathway (Liu et al., 2012). In addition to using WGA as a targeting ligand for nanoparticles, many lectins have been shown to modify the surface of nanocarriers to facilitate nose-to-brain delivery, such as Eggplant tuber lectin, fluorescent probes, and basic fibroblast growth factor (bFGF) (Lundh et al., 1989; Chen et al., 2012; Zhang et al., 2014). The glycoprotein lactoferrin (Lf), the ligand of the lactoferrin receptor (LfR) is highly expressed in brain neurons and is the most commonly used nanocarrier-targeting ligand (Huang et al., 2008). In addition, Lf-functionalized nanoparticles have a stronger neuroprotective effect than other nanoparticles (Liu et al., 2013). Several other ligands are also used to modify nanoparticles, including rabies virus glycoprotein (RVG29) and inorganic nanoparticles (Gallardo-Toledo et al., 2020; Hao et al., 2020).

## 4 Factors influencing the intranasal administration of drugs into the brain

Drug delivery to the brain via intranasal administration, whether through indirect or direct routes, requires an effective drug dose to reach and cross the mucosal and epithelial of the nasal cavity (Bicker et al., 2020). Effectively overcoming this difficulty is the key to successful drug delivery. Various factors influence the intranasal administration of drugs, including the drug selected, the therapeutic indication, the individual population, and the delivery device (Misra and Kher, 2012). Nevertheless, these factors cannot be separated from the nature of the drug itself and the state of the nasal cavity. Therefore, this section discusses these two aspects in the selection of appropriate nanocarriers.

### 4.1 Properties of the nanocarriers

#### 4.1.1 Size and morphology

Molecular weight is the main limiting factor for drug absorption (Ozsoy et al., 2009), and there is an inverse relationship between the drug particle size and the degree of effective nasal absorption of that drug (Henkin, 2010). Nanocarriers <200 nm can usually deliver drugs to the brain via extracellular pathways, and nanocarriers 20–100 nm are easily transported via extracellular pathways and along axons, where the permeability of 20-nm drugs is twice as high as that of 100-nm drugs (Brooking et al., 2001). In addition, nanoparticles with larger sizes are cleared by sneezing (Gänger and Schindowski, 2018). The drug morphology also affects absorption in the nasal cavity; cyclic particles are more readily absorbed than linear particles (McMartin et al., 1987), and polymorphic forms are better solubilized, thus easily passing through biological membranes (Morissette et al., 2004).

#### 4.1.2 Surface charge

Drugs with a cationic surface charge can be more easily administered through the nasal cavity (Tan et al., 2020). On the one hand, this surface charge can disrupt tight junctions for better transport through extracellular pathways. On the other, nasal secretions, as many anionic links, can attract drug binding for better transport.

#### 4.1.3 Lipophilicity

**FIGURE 6:**
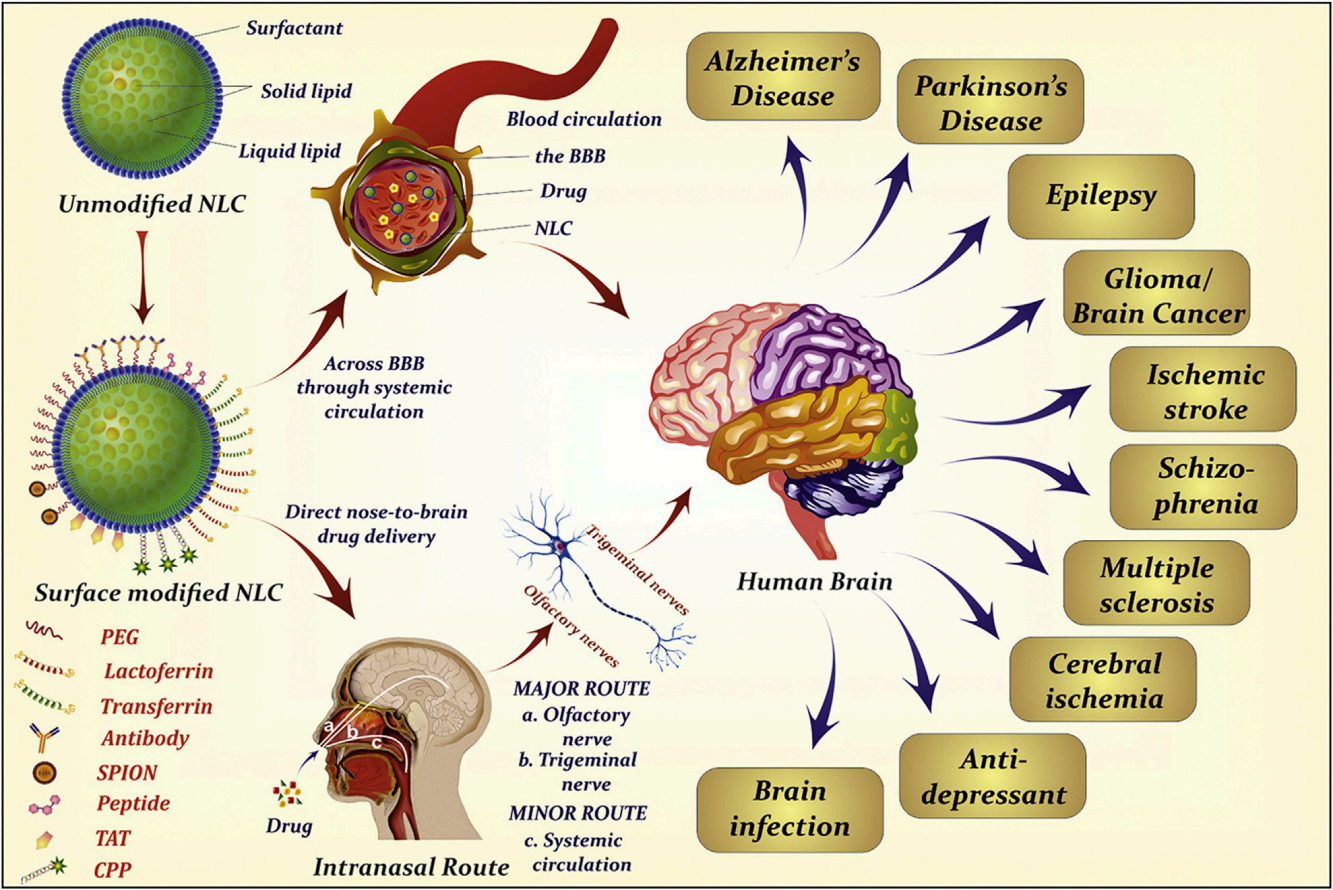
Extensive use of lipid nanoparticles in neurodegenerative diseases. Reproduced with permission (Agrawal et al., 2020b). Copyright 2020, Elsevier.

Drug surface chemistry determines the main transport pathway. Although a few hydrophilic properties exist, the nasal mucosa are mainly lipophilic. The use of lipophilic drugs (e.g., lipid nanoparticles) can better aid intracellular transport (Figure 6) (Agrawal et al., 2020b), while hydrophilic drugs can aid extracellular transport (Clementino et al., 2021). Usually, when a drug’s lipophilicity increases, the drug’s dose penetrating the nasal mucosa increases.

### 4.2 Physiological factors of the nasal cavity

#### 4.2.1 Mucosal mucociliary clearance and cilia movement

Nasal mucociliary clearance (MCC) is an important physiological mechanism for the nasal clearance of foreign bodies, including bacteria, dust, allergens, and secretions. The rate of MCC is 1–2 mm/h in the anterior inferior turbinate and 8–10 mm/h in the posterior inferior turbinate (Jones, 2001). Thus, placing the drug in the anterior inferior turbinate can increase drug delivery efficiency. MCC can completely clear liquid formulations in 12 min (Shaikh et al., 2011), and swallowing can drain the formulation from the nasopharynx to below; thus, drug can be lost from liquid formulations and affect the gastrointestinal tract. Within a certain range, as the temperature increases, the mucosal cilia movement also increases, and at ∼24 °C, the MCC decreases, facilitating drug retention and absorption (Corbo et al., 1990).

#### 4.2.2 Intranasal secretions

The nasal mucosa and submucosal glands secrete mucus that is continuously present on the nasal mucosa in a bilayer and has a thickness of approximately 5 µm (Arora et al., 2002), with an aqueous layer at the bottom, in which the epithelial cilia move, and a viscous gel layer at the top, in which the gel-like surface follows the cilia forward. The viscosity of the secretion determines the drug retention time, and the composition of the secretion determines the ease of drug dissolution. Nasal secretions comprise 90% water, with the rest comprising mucin, salts, proteins, and lipids (Arora et al., 2002). Drugs with similar physicochemical properties to nasal secretions aid absorption, and studies have shown increased absorption of drugs when aqueous analogs are given intranasally (Kao et al., 2000). Reportedly, changes in the viscosity of secretions can affect cilia movement and alter the drug retention time, affecting drug absorption (Mortazavi and Smart, 1994); for example, anionic drugs enhance mucus gelation more so than neutral or cationic drugs, thereby increasing drug retention and enhancing absorption.

#### 4.2.3 Nasal cycle

It has been shown that the nasal cycle occurs less frequently at night than in the daytime (Merkus et al., 1998). As a result, cilia movement of the nasal mucosa is reduced at night, as is the secretion production and clearance rate, influencing drug absorption.

### 4.3 Pathological states

Nasal diseases, such as acute and chronic rhinitis, allergic rhinitis, drug rhinitis, and nasal polyposis, usually alter the normal physiological state of the nasal mucosa, resulting in insufficient or excessive mucus secretion, swelling or drying of the mucosa, altering the conditions of drug absorption, leading to reduced drug absorption (Gizurarson, 1993). In addition, pruritus and sneezing caused by rhinitis can exacerbate this effect, and drug administration is greatly diminished in these states. Nasal secretions are acidic, and when the pKa of the drug is higher than the pH of the nasal secretions, the drug does not ionize but exists intact and is more easily absorbed (Huang et al., 1985). Infections can change the pH of nasal secretions. For example, chronic bronchitis can increase the pH of nasal secretions to 7.8, affecting the drug’s pH and thereby ionizing the drug and limiting absorption (Adler et al., 1972). The ideal pH of the nasal secretions for drug delivery is 4.5–6.5; the drug can somewhat counteract this pH change.

### 4.4 Nasal microbiota

The complex microbiota in the nasal cavity, including bacteria, fungi, and viruses, has a complex relationship with the host with different compositions (Dominguez-Bello et al., 2019). Nasal microbiota can affect the olfactory nerve or lymphatic drainage, leading to the occurrence and development of NDs (Bell et al., 2019). Reportedly, the nasal microbiota has both pathogenic and therapeutic effects on NDs (Xie et al., 2022). Therefore, selecting drugs compatible with the individual microbiota allows for better treatment.

### 4.5 Nasal immune system

Nasal mucosal epithelial cells secrete various enzymes that affect the stability of intranasal drugs, especially peptide and protein drugs, which are broken down by endopeptidases, such as serine, and exopeptidases, such as mono and diamino peptidase (Kaur et al., 2016). In addition, the nasal cavity contains immunoglobulins such as IgS, which can combine with peptide drugs to form complexes (Kakad et al., 2015), making it difficult for the drug to pass through the nasal mucosa if the size exceeds 500 nm. Therefore, it is critical to effectively package the drug to minimize the effect of the immune system on the drug.

## 5 Strategies for enhancing nasal-brain delivery

### 5.1 Absorption enhancers

Tight junctions in the nasal cavity’s olfactory and respiratory epithelial regions (Ruigrok and de Lange, 2015) protect the internal mucus layer and limit the passage of drugs through the nasal mucosa. Absorption enhancers are compounds delivered via intranasal administration along with the drug and can temporarily alter the structure of the nasal mucosa, opening the tight junctions between cells, allowing easier passage, and protecting the drug from degradation (Kim et al., 2020). In addition, the lipophilic and cationic properties of the drug can better expand the effect of crossing the nasal mucosa. Enterotoxin is a good absorption enhancer but may cause side effects, such as tissue damage and reduced cilia movement, which can be reduced using surfactants, protease inhibitors, and tight junction modifiers (Ghadiri et al., 2019). Cationic polymers such as chitosan and its derivatives can act on the nasal mucosal barrier to alter tight junctions, thus improving drug absorption (Ghadiri et al., 2019). Surfactants such as bile salts, nonionic surfactants, phospholipids, and fatty acid salts can better help drugs cross the nasal mucosa by filtering out membrane proteins, opening tight junctions, or preventing enzymatic drug degradation (Ghadiri et al., 2019). Encapsulation of the impermeable drug flurbiprofen in nanoparticles can lead to the better passage of drugs for AD through the endothelial cell monolayer, reducing Aβ42 levels and modulating γ-secretase activity (Meister et al., 2013).

Notably, CPPs can promote biomolecular and cellular internalization (Said Hassane et al., 2010), which would be an interesting strategy for promoting drug uptake. Transactivated transcription (TAT) peptides deliver siRNA to the rodent brain via olfactory nerve pathways to treat AD (Kim et al., 2016). CCPs with hydrophobic (stearate) or hydrophilic (polyethylene glycol) modifications can target relevant drugs to different sites of action in the brain via olfactory pathways (Kanazawa et al., 2017).

### 5.2 Enzyme inhibitors

The nasal mucosa contains various enzymes that target and degrade delivered drugs, including transferases, carboxylesterases, and peptidases (Agrawal et al., 2020a). Amylosucrase inhibits aminopeptidase, while bacitracin and puromycin inhibit the protection of leucine enkephalin from enzymatic degradation. Disodium EDTA protects β-fold disrupting peptide protection from enzymatic degradation for better treatment of AD (Laffleur and Bauer, 2021).

### 5.3 Prodrugs

Prodrugs are used to overcome poor solubility, poor chemical or biological stability, poor absorption, and premature metabolism by transiently modifying the binding of specific functional groups of the drug to the precursor fraction to make it favorable for nasal mucosal absorption (Tirucherai et al., 2001). When the drug passes through the nasal mucosal barrier, this transient modification can avoid drug loss and thus allow optimal drug bioavailability. Nevertheless, the side effects of prodrugs for treating AD must be considered; for example, inhibiting cholinesterase increases acetylcholine in the brain (Blanco-Silvente et al., 2017), causing diarrhea, vomiting, and nausea and affecting the cognitive aspects of the patient’s treatment (Loy and Schneider, 2006).

### 5.4 Vasoconstrictors

The nasal respiratory region has a high density of vascular distribution and is the main target for systemic drug delivery. The olfactory epithelium is less vascular and is the main target for nerve pathways and nose-to-brain delivery. A study on intranasal delivery of neuropeptide and hypocretin-1 showed that intranasal administration increased the drug dose delivered to the olfactory bulb after vasoconstrictor administration (e.g., phenylephrine) and decreased the amount of drug in the circulation (Dhuria et al., 2010).

### 5.5 Drug delivery devices

The key to the success of intranasal drug delivery targeting the brain is to maximize the deposition of the drug formulation in the olfactory epithelial. Today, the most commonly used drug delivery devices are nasal drops and pump sprays. Nasal drops diffuse widely in the nasal cavity, increasing the deposition area in the olfactory epithelial (Hardy et al., 1985); with mucoadhesion, the retention time is up to 14 min (Charlton et al., 2007). However, applying nasal drops is complex and requires good patient administration technique and proper head position (Vidgren and Kublik, 1998). Pump sprays are simple to operate and can deliver a constant and stable dose of 25–200 mL (Vidgren and Kublik, 1998). Plume and delivery angles are key determinants of deposition efficiency, with maximum deposition efficiency to the turbinate (30%–50%) occurring at a plume angle of 55–65°; an approximately 90% deposition efficiency can be achieved using a 30° delivery angle (Foo et al., 2007). A deposition of 2.5% occurred in the area corresponding to the olfactory region (Djupesland and Skretting, 2012).

Vianase is an electronic nebulizer developed by Kurve Technology in which the nebulized drug moves in a vortex chamber and maintains this motion after leaving the device, maximizing drug deposition in the olfactory region (Warnken et al., 2016). This device has been used to deliver insulin to the brain to treat AD (Craft et al., 2012) and reached the patient’s brain within 2 min. However, some pulmonary complications were observed with long-term use (Rapoport and Winner, 2006). Therefore, the safety of nasal sprays in the context of long-term treatment is unclear.

## 6 Nose-to-brain delivery for specific NDs

Intranasal drug delivery is becoming an effective route for treating many NDs such as AD, PD, HD, and prion diseases (Chapman et al., 2013). These diseases are usually slowly progressive (Soto and Pritzkow, 2018) and are characterized by the misfolding and accumulation of intra-and extracellular protein deposits and neuron disorders, apoptosis, or necrosis, mainly manifesting as motor, cognitive, and mental impairments. Therefore, treatment involves pharmacological agents that counteract the disease pathogenesis and symptoms. Here, we summarize the application of nanocarriers for nose-to-brain drug delivery in different NDs (Table 2).

**TABLE 2:** Nanocarriers in neurodegenerative diseases.

Disease	Strategy	Nanocarrier	Drug loading	Experimental subjects	Results	Reference (DOI)
AD	**Mucoadhesion**	Cationic biopolymer nanoparticles	Lutein	Male Sprague Dawley (SD) rats	Higher penetration rates than neutral or negative nanoparticles	10.1016/j.ijpharm. 2020.119553
		Polymer nanoparticles	Diphtheria toxoid	Amyloid β (Aβ) rats	Reducing AD memory impairment	10.1007/s43440-019-00017-w
		Thiolated chitosan nanoparticles	Galanthamine	Swiss male albino mouse	Significantly improving acetylcholinesterase levels in mouse brain	10.1007/s13311-018-00694-0
		Chitosan nanoparticles	Galanthamine hydrobromide	Male Wistar rats	Reducing AChE levels and activity and the hyperphosphorylation of interleukin and tau in the brain	10.3109/10717544.2016.1153748
		Polymeric nanoparticles and solid lipid nanoparticles	Tarenflurbil	Male SD rats	Nanomedicine can be transported directly to the brain via olfactory nerve pathways	10.1016/j.ejps. 2016.05.012
		Polyethylene glycolic liposomes	Rivastigmine	Male albino rabbits	The bioavailability of drug at plasma and brain levels increased to 4-fold	10.1080/10717544.2017.1309476
		Liposomes	H102 (novel β-sheet breaker peptide)	Male SD rats	Improved spatial memory deficits in rats, increased cholinesterase levels and decreased the number and size of Aβ plaques	10.1007/s11095-015-1744-9
		Nanoemulsions	Memantine	Male SD rats	Encapsulation of mevalonate in nanoemulsions maintained its antioxidant potential	10.1080/02652048.2020.1756971
		Nanoemulsion of chitosan nanocapsules	P38MAPK inhibitor	Male Wistar rats	Reducing p38MAPK phosphorylation in the brain	10.2217/nnm-2018-0496
		Nanoemulsion	Donepezil	Male SD rats	A new method for treating AD via nose-to-brain drug delivery	10.1007/s13346-020-00754-z
	**Microspheres**	Polymeric nasal microspheres	Hydroxypropyl-β-cyclodextrin	Male SD rats	Oxidative stress and apoptosis are inhibited in the rat hippocampus, with protective effects against β-Amyloid (1-42)-induced neurotoxicity	10.1016/j.xphs. 2016.05.017
		Lectin-functionalized microspheres	Rivastigmine	Male Wistar rats	Better memory retention in rats	10.1016/j.biopha. 2021.111829
		Microemulsion	Galanthamine hydrobromide	Male SD rats	Penetrating the BBB more easily and enhancing efficacy against oxidative stress	10.1007/s13346-020-00739-y
		Microemulsion	Rivastigmine tartrate	Male SD rats	Reversible increase of rivastigmine tartrate in the brain	10.1007/s11095-017-2279-z
		Microemulsion	Morin hydrate	Male Wistar rats	Treating memory of AD rats on day 21	10.1080/21691401.2016.1276919
	**Stimulus-responsive drug carriers**	Thiolated chitosan hydrogel	Donepezil	Rabbits	Increasing the mean brain content of the drug	10.1038/s41598-019-46032-y
		*In situ* gel nanostructured lipid carrier	Resveratrol	Male SD rats	Enhanced memory function and nasal mucosal permeation in rats	10.1007/s13346-018-0540-6
		Trimethyl chitosan hydrogel nanoparticles	Progesterone	Male SD rats	A 5-fold increase in brain progesterone concentration after 30 min of inhalation of hydrogel nanoparticles	10.3390/pharmaceutics11120657
	**Targeted and functionalized nanocarriers**	Wheat germ agglutinin nanoparticles	miRNA 132	APP/PS1 double transgenic mice	Reducing Aβ protein and improving learning and memory functions	10.3389/fphar. 2020.01165
		Lactoferrin-coupled trimethylated chitosan	Huperzine A	Male Kunming (KM) mice	Higher fluorescence intensity and longer residence time in the brain	10.2147/IJN.S151474
		Lactoferrin-modified nanoemulsions	Huperzine A	Male Wistar rats	Higher brain targeting than unmodified nanoemulsions	10.2147/IJN.S214657
PD	**Mucoadhesion**	Thiolated Polymer (nasal dry powders)	-	RPMI 2650 cells	Enhancing mucoadhesion when used *in vitro*	10.1016/j.ijpharm. 2022.122188
		Chitosan nanoparticles	Pramipexole	Male SD rats	Better local neurological function, enhanced antioxidant status, and increased dopamine levels in the brain	10.1016/j.ijbiomac. 2017.12.056
		Chitosan nanoparticles	Rasagiline	Male Swiss albino mice	Enhanced bioavailability in the brain	10.3109/10717544.2014.907372
		Nanoparticle	P substance	6-hydroxydopamine (6-OHDA)-induced PD rats	6-OHDA-induced apoptosis was inhibited in PD rats	10.2147/DDDT.S77237
		Trimethyl chitosan-modified nanoemulsion	Ropinirole-dextran sulfate	Female Swiss albino mice	High brain targeting efficiency via olfactory pathway	10.1016/j.ijbiomac. 2018.09.032
		Solid lipid nanoparticles	Geraniol/ursodeoxycholic acid	Rats	Facilitating the entry of geraniol and ursodeoxycholic acid into the brain at low doses	10.1016/j.jconrel. 2020.02.033
	**Targeted and functionalized nanocarriers**	PLGA combined with lectin	Levodopa	Male CD57/BL6 mice	Enhancing nasal absorption of nanoparticles with better targeting ability and lower cytotoxicity	10.1080/10837450.2020.1740257
		Borneol and lactoferrin co-modified nanoparticles	Dopamine	Male SD rats	Promoting cellular uptake of nanoparticles and higher brain drug concentrations within 12 h	10.1080/10717544.2019.1636420
		Lactoferrin-modified PEG-PLGA nanoparticles	Rotigotine	Mice	High drug accumulation in the striatum after intranasal administration	10.2147/IJN.S120939
		Angiopep-2 and polysorbate 80-modified liposomes	Cyclovirobuxine D	Male SD rats	Easily crossing the BBB with high drug concentrations in the brain	10.1166/jbn. 2018.2581
HD	**Mucoadhesion**	Liposomes	Exogenous brain cholesterol	Wild-type (WT) mice	Effectively delivering cholesterol to the brain via olfactory and trigeminal nerve pathways	10.1021/acschemneuro.9b00581
		Solid lipid nanoparticles	Rosmarinic acid	Male Wistar rats	Reduced deficits in walking ability, locomotion, and motor coordination transitions in rats, oxidative stress in the striatum, and unnecessary drug metabolism in other parts of the body	10.3109/10717544.2014.880860
		Chitosan nanoparticles	siRNA for HTT	YAC128 transgenic mice	Reducing the expression of the erroneous gene HTT mRNA by more than 50%	10.1016/j.jddst. 2021.102517

### 6.1 Alzheimer’s disease

**FIGURE 7:**
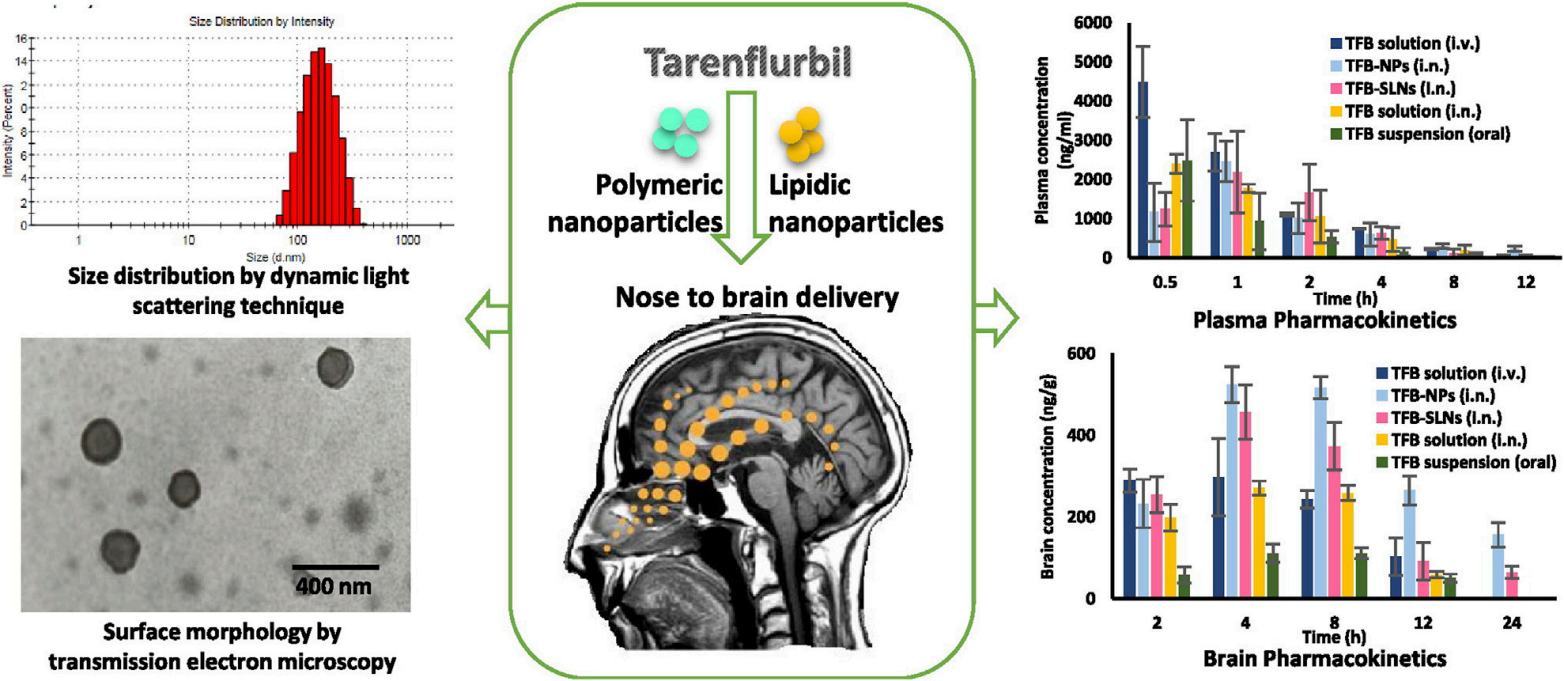
Nanoparticle-encapsulated tarenflurbil for AD. Reproduced with permission (Muntimadugu et al., 2016). Copyright 2016, Elsevier.

**FIGURE 8:**
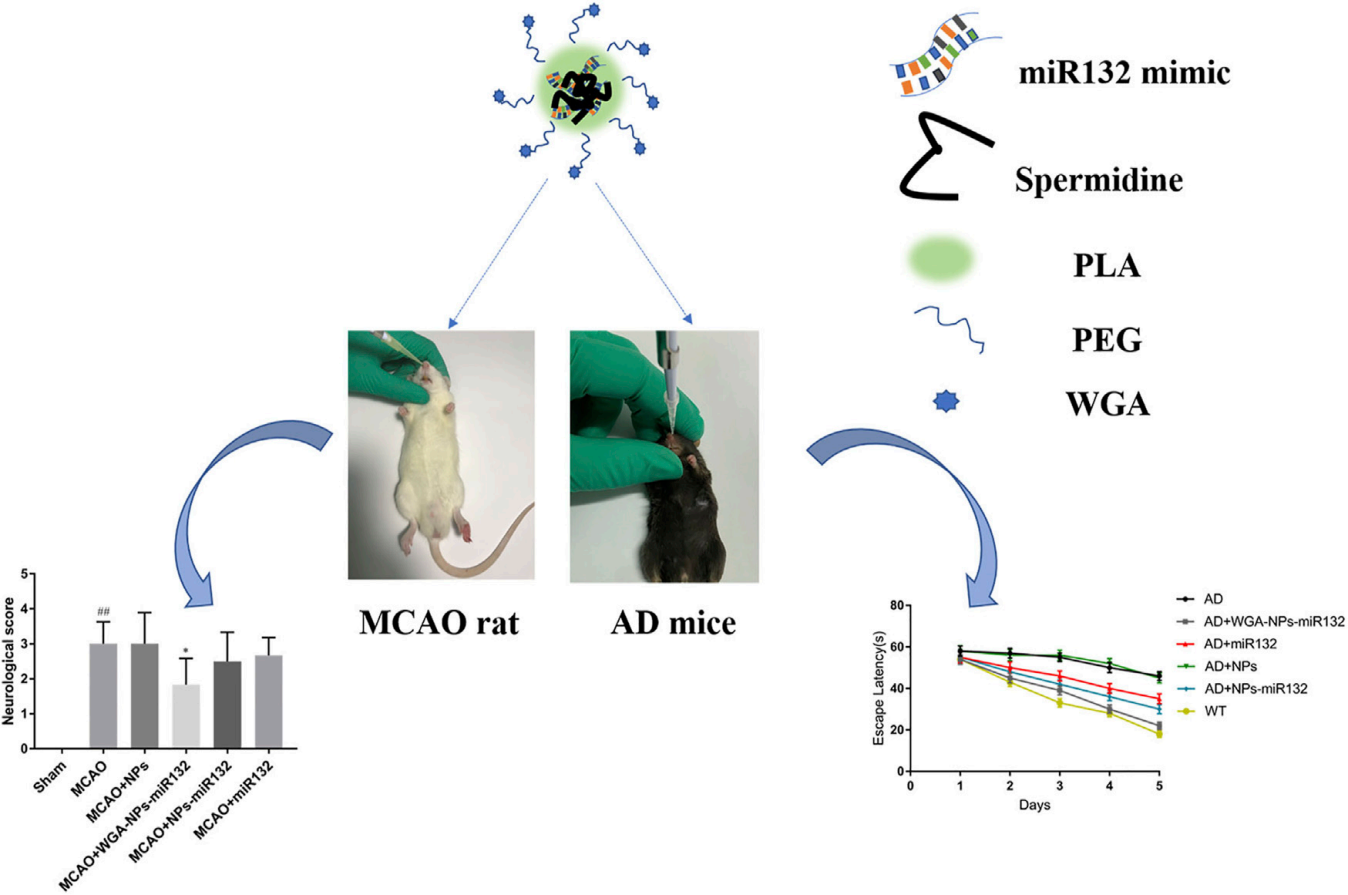
Intranasal delivery (WGA)- nanoparticles -miR132 for AD. Reproduced with permission (Yu et al., 2020). Copyright 2023, Frontiers Media S.A.

AD is the most common ND (Breijyeh and Karaman, 2020) and the most common cause of dementia, with a projected global prevalence of 150 million in the mid-21st century. The main symptoms of AD are cognitive behavioral loss, memory impairment, and impairment in daily activities. AD pathogenesis is mainly divided into positive and negative lesions. Positive lesions are mainly caused by abnormal protein accumulation inside and outside brain cells, including neurofibrillary tangles, amyloid plaques, and other deposits. Amyloid β (Aβ) protein deposition or Aβ plaque formation is one of the major biomarkers of AD, and tau is another important biomarker. Hyperphosphorylation of tau can cause neurofibrillary tangles (NFTs). Negative lesions are mainly atrophy of brain areas due to the loss of neurons and synapses. In addition, cholinergic system dysfunction and oxidative stress can cause AD. Therefore, the treatment is based on cholinesterase inhibitors, neuron death reduction, deposit accumulation prevention, and antioxidants (Figures 7, 8). Thiolated chitosan (modified) nanoparticles were fabricated using modified ionic gelation method in one study. Galantamine loaded thiolated chitosan nanoparticles were showed to lead to better efficacy than conventional oral therapies (Sunena et al., 2019). Similarly, in three other studies (Muntimadugu et al., 2016; Jiang Y. et al., 2019; Yu et al., 2020), poly (lactide-co-glycolide) or solid lipid nanoparticles loaded with tarenflurbil (TFB), Wheat germ agglutinin (WGA) -modified PEG/PLA nanoparticles loaded with miR-132 (Muntimadugu et al., 2016; Yu et al., 2020) and lactoferrin loaded uperzine A (HupA) nanoemulsion (Jiang Y. et al., 2019) showed significant benefits for AD.

### 6.2 Parkinson’s disease

**FIGURE 9:**
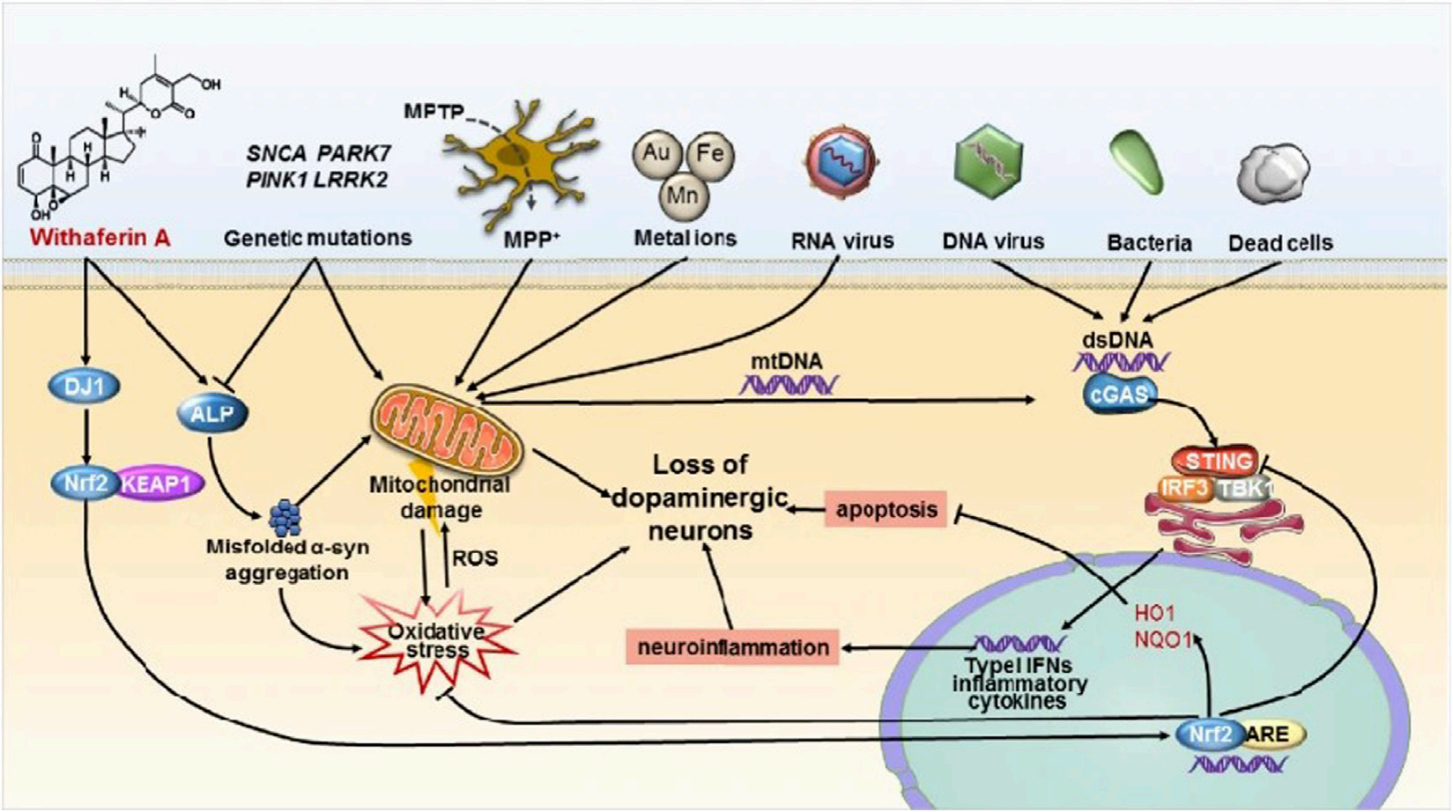
STING-mediated neuroinflammation-induced apoptosis may be a novel mechanism in Parkinson’s disease. Reproduced with permission (Zhao et al., 2021). Copyright 2021, Springer Nature.

**FIGURE 10:**
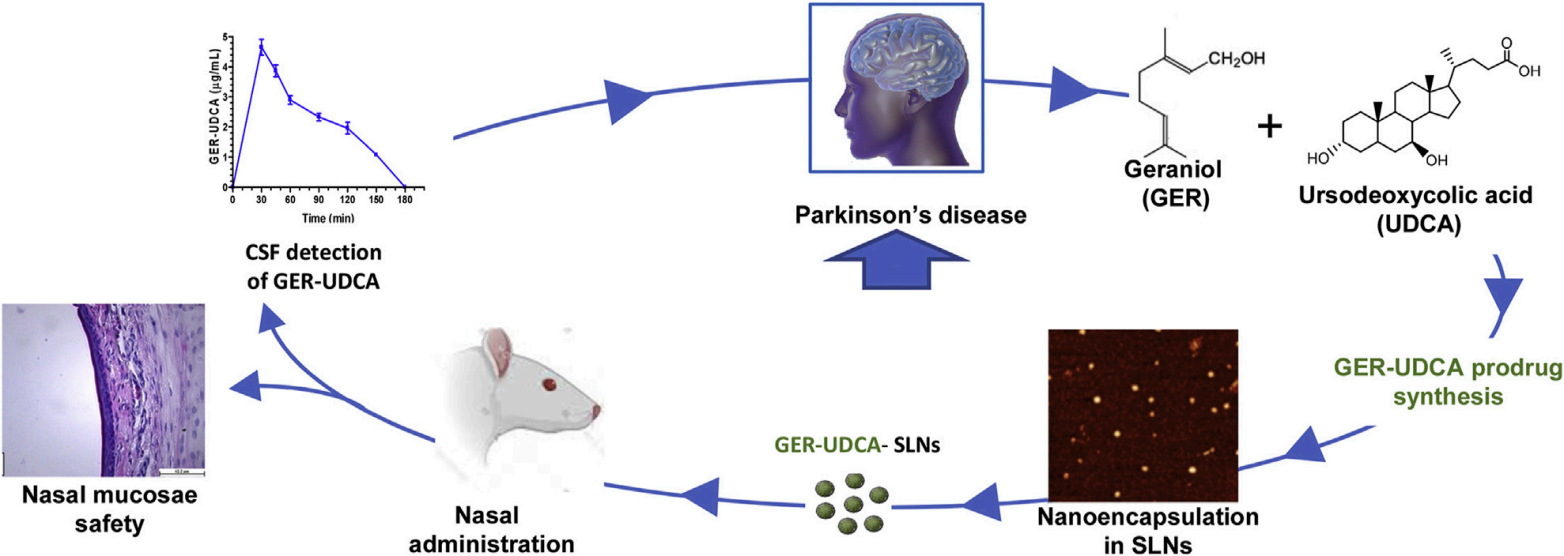
Nanoparticle-encapsulated geraniol/ursodeoxycholic acid coupling for PD. Reproduced with permission (de Oliveira Junior et al., 2020). Copyright 2020, Elsevier.

PD is the second most common ND (Beitz, 2014). The main symptoms are motor disorders, such as bradykinesia, tonicity, and tremor, and some nonmotor disorders, such as cognitive impairment and behavioral disturbances. The pathogenesis of PD is the loss of dopaminergic neurons at the substantia nigra. The deposition of Lewy bodies in the brain leads to impairment of neurotransmitter systems, such as uncontrolled excitation of cholinergic neurons and γ-aminobutyric acidergic neurons of the corpus striatum, leading to dyskinesia (Figure 9) (Zhao et al., 2021). Therefore, treatment is based on supplying dopamine or restoring the dopamine transmission system with levodopa as a precursor (Figure 10) (de Oliveira Junior et al., 2020). The nanoencapsulated geraniol/ursodeoxycholic acid conjugate constructed by the researchers was proven to be an effective strategy and did not damage the structural integrity of the nasal mucosa in contrast to the pure geraniol (de Oliveira Junior et al., 2020).

### 6.3 Huntington’s disease

**FIGURE 11:**
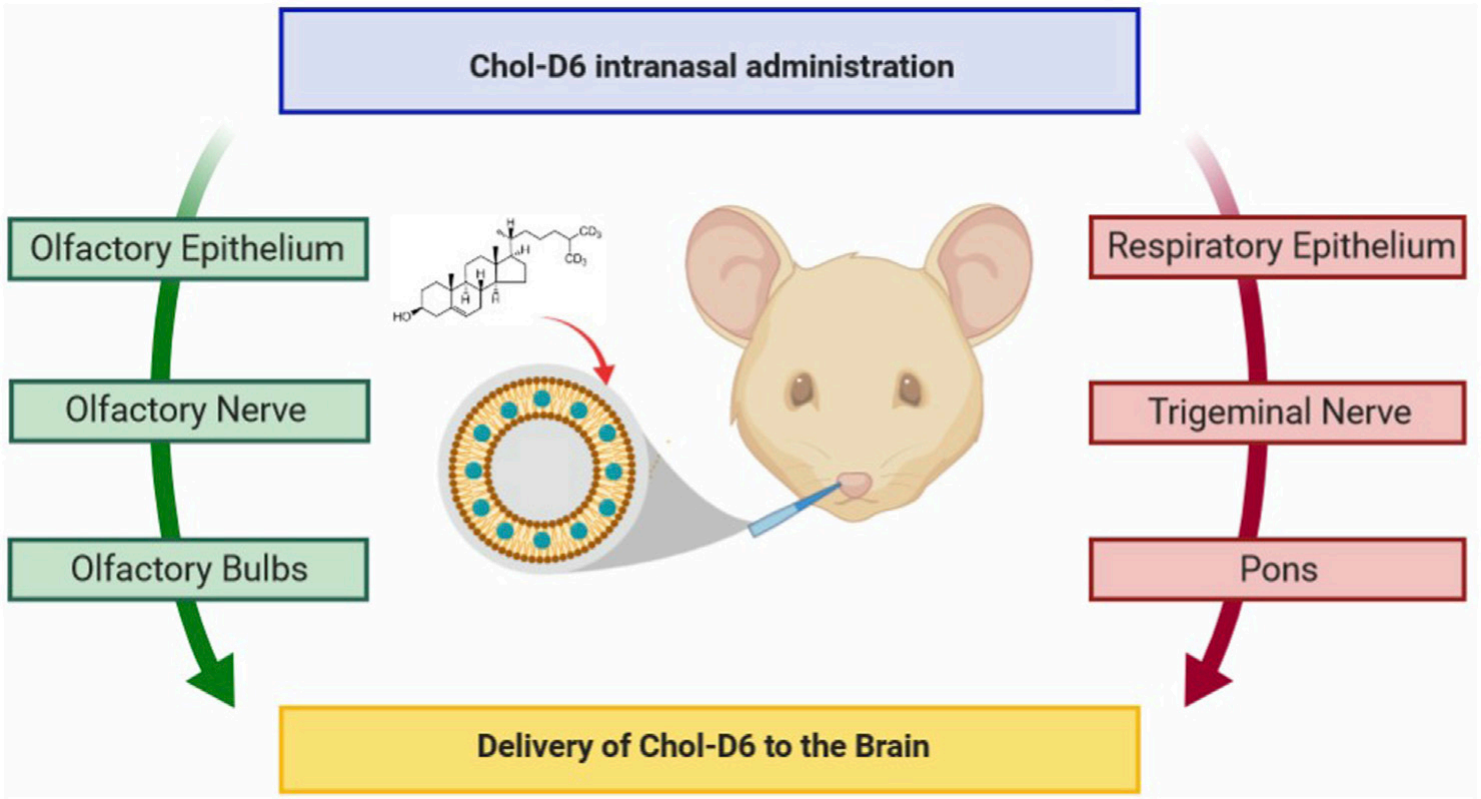
Cholesterol delivery by the nose-to-brain pathway for HD. Reproduced with permission (Passoni et al., 2020). Copyright 2020, American Chemical Society.

HD is a relatively rare ND (Stoker et al., 2022). Characterized by motor deficits and cognitive decline with some psychiatric symptoms such as depression and anxiety, HD is mainly due to abnormal duplication of CAG nucleotides of the gene encoding the Huntington protein, which eventually produces abnormal protein polyglutamine (polyQ) and largely accumulates in the neurons of basal ganglia (caudate-putamen), and finally cortico-striatal dysfunction. Treatment for HD is often palliative, and neurotrophic factors are critical (Figure 11) (Passoni et al., 2020). The constructing intranasal cholesterol-loaded liposomes (IN Chol-loaded liposomes) has not been shown to improve efficacy (Passoni et al., 2020).

### 6.4 Sensorineural deafness

Sensorineural hearing loss (SNHL) is a group of neurodegenerative disorders associated with hearing loss (Park et al., 2018), the main cause of which is irreversible damage to the cochlear hair cells, ultimately leading to the decline of spiral ganglion cells. Sensorineural deafness is the main type. An interconnection between dementia (e.g., AD and other cognitive impairment disorders) and hearing loss has been demonstrated (Peters et al., 1988). Deafness is a common feature of aging- and dementia-related disorders, which are associated with central auditory pathways and brain lesions, such as reduced scavenging of oxygen free radicals, mitochondrial DNA damage, and enhanced deamidation and ubiquitination (Lindner and Helliger, 2001; Chondrogianni and Gonos, 2005; Schaar et al., 2015). One study found that these two disorders significantly increased amyloid precursor protein and phosphorylated tau in the cerebral cortex (Park et al., 2018). Therefore, in addition to specialist treatments, such as hearing aids, other medications may overlap with the treatment of AD, such as antioxidant therapy and medications to reduce neuronal death and prevent deposit accumulation. Nanohydrogel may be used for inner ear dialysis, a promising treatment for SNHL (Li et al., 2017).

## 7 Opportunities and challenges

The anatomical, physiological, and nasal dynamics of nasal drug delivery to the CNS remain challenging. The low volume of the nasal cavity limits the dose administered, which, combined with the mucosal ciliary clearance of the nasal cycle, largely affects the absorption rate. Various enzymes, such as proteases, endopeptidases, and carboxypeptidases, degrade drug proteins and peptides during the limited retention time of the drug. However, with the advent of nanotechnology, these unfavorable drug properties no longer limit intranasal drug delivery by modulating the delivery vehicle rather than the nature of the drug. These problems can be overcome by rational drug delivery strategies and delivery methods, and these adverse drug properties no longer limit intranasal drug delivery.

Gene therapy can achieve neuroprotection and neurorepair and ultimately correct pathogenic mechanisms rather than alleviate the symptoms of NDs (Sudhakar and Richardson, 2019). *In vivo* siRNA or ASO delivery is effective in animal models of neurodegenerative pathologies such as AD, PD, and HD (Sah, 2006; Sudhakar and Richardson, 2019). Viral vectors account for over 70% of all active gene therapy clinical trials; however, invasive delivery systems are required for treating neurological disorders, such as stereotaxic injection of viral vectors into the brain, which is highly impractical (Dreyer, 2011). In addition, repeated injections into multiple brain regions are unacceptable for AD, PD, and HD as these pathologies gradually spread throughout the brain. Invasive injections are inherently risky, causing bleeding and infection. The intranasal delivery of molecules from the nasal cavity to the olfactory bulb to the brain is feasible (Lochhead et al., 2015; Aly et al., 2019) and allows for the noninvasive delivery of molecules to the brain, bypassing the BBB; thus, this approach can be used to carry nonviral-vector gene therapies for NDs (Villatebeitia et al., 2015). Using nanocarriers for gene delivery allows the simple, noninvasive, and chronic administration of drugs to patients. Several studies have been conducted on nanocarrier gene delivery in recent years (Kanazawa et al., 2013; Fan et al., 2018; Sanchez-Ramos et al., 2018; Sava et al., 2020; Sava et al., 2021; Alamoudi et al., 2022; Petkova et al., 2022). Therefore, the intranasal delivery of nanocarriers will have a significant impact on the treatment of brain diseases in the future.

The efficiency of nose-to-brain delivery is mainly assessed via neural pathways located in the posterior and apical olfactory and the respiratory epithelium of the nasal cavity, which are difficult to access and deposit (Quintana et al., 2016). Furthermore, no studies have guided the use of nasal sprays as a delivery device for systemic absorption after intranasal administration, and mucosal inflammation of the nose, nasal polyps, and deviation of nasal septum affecting the deposition of sprays have not been explored.

Nanoparticles as carriers for nose-to-brain delivery of drugs must be studied in more depth for use in clinical applications, including aggregation and clearance due to nanoparticle size, resulting in toxicity with control and clearance. Similarly, the potential toxicity of nanoparticles to the nasal mucosa and brain remains unclear. Each nanocarrier’s contribution is unknown, and the co-dosing association must be studied. The selection of excipients to reduce drug toxicity also awaits study in the development of drug delivery systems.

## 8 Conclusion

This review compared different nanomedicines and summarized the methods, influencing factors, and strategies for enhanced delivery of nanomedicines based on nasal targeting to the brain and their relevant applications in treating NDs. After intranasal administration using appropriate methods, the therapeutic efficacy of these nanomedicines has demonstrated good results in several studies. However, the clinical application of nanotechnology delivery systems for nose-to-brain drug delivery is in the early stages of development. Conclusive studies in additional animal models (e.g., monkeys) are necessary to elucidate the appropriate characteristics that nanoparticles must possess to serve as successful nose-to-brain drug delivery vehicles to facilitate this translation to the clinic. In addition, more conclusive data on the pharmacodynamics and pharmacokinetics of the different routes and the biodistribution of biomolecules in the brain are needed.
