# Supplementary material for: Transnasal-brain delivery of nanomedicines for neurodegenerative diseases
Source: Front Drug Deliv. 2023 Aug 11;3:1247162. doi: 10.3389/fddev.2023.1247162 (PMC12363324; doi:10.3389/fddev.2023.1247162)
Supplement: Supplementary file 1 [file Table1.DOCX]

**Table 1.** Comparison of the advantages and disadvantages of nanocarriers for nose-to-brain drug delivery

| **Nanocarriers** | **Properties** | **Advantages** | **Disadvantages** |
| --- | --- | --- | --- |
| Polymeric nanoparticles | Polymer | 1. Combination of various, more stable than single-molecule carriers  2. Can deliver multiple substances with high loading capacity  3. Ligand-modified, targetable, anti-degradation, low-toxic | 1. Instant drug release, not sustained drug release  2. High cost, difficult to produce on a large scale. |
| Lipid nanoparticles | For delivery of lipophilic drugs | 1. Wide range of raw materials, easy to produce  2. High biocompatibility and safety  3. Controlled drug release for a long time  4. Large surface area and high ability to penetrate the BBB | 1. Unstable, easily degraded  2. Difficult to package water-soluble substances  3. Weak ability to release drugs instantly |
| Micelles | Assembled from lipid nanoparticles and core surfactant | Smaller than other nanoparticles | 1. Less biocompatible than lipid nanoparticles  2. Potential toxicity of the core surfactant |
| Nanoemulsions | Liquid lipid | 1. Can be made into a spray for easy drug delivery  2. Mucoadhesion to increase drug retention time in the nasal cavity to improve drug bioavailability | Requiring a large amount of surfactant to prepare makes the drug ineffective when passing through biofilm |
| Nanogel | Crosslinked polymer network | 1. High hydration, high deformation ability, high loading capacity to deliver multiple drugs  2. High ability to penetrate the BBB and continuous drug release | 1. Unstable integration, acid-sensitive and reductive, easily degraded  2. Component materials can cause some damage to the nasal cavity |
| Cell-penetrating peptide | Compounded with selected drugs | 1. Penetrating cell membranes with high biocompatibility and safety;  2. High ability to penetrate BBB;  3. targetable | 1. Unstable  2. Can enter all cells with toxicity and lower drug concentration at the specified site |
| Inorganic nanoparticles | Inorganic material | 1. Specified material composition, easy to produce, more stable compared to organic materials  2. Mesoporous inorganic nanoparticles can be targeted, high loading capacity and biocompatibility, easy diffusion in the brain | 1. Difficult to remove and toxic  2. Uncontrolled drug release |
| Exosomes | A single lipid membrane vesicle secreted by cells | 1. Can be fused with cells with high biocompatibility and safety 2. Readily crosses the BBB   3. Can be used in multiple ways to treat NDs with high potential | Difficulty in targeting delivery |
