# Supplementary material for: Transnasal-brain delivery of nanomedicines for neurodegenerative diseases
Source: Front Drug Deliv. 2023 Aug 11;3:1247162. doi: 10.3389/fddev.2023.1247162 (PMC12363324; doi:10.3389/fddev.2023.1247162)
Supplement: Supplementary file 2 [file Table2.DOCX]

**Table 2.** Nanocarriers in neurodegenerative diseases

| **Disease** | **Strategy** | **Nanocarrier** | **Drug Loading** | **Experimental subjects** | **Results** | **Reference**  **(DOI)** |
| --- | --- | --- | --- | --- | --- | --- |
| **AD** | **Mucoadhesion** | Cationic biopolymer nanoparticles | Lutein | Male Sprague Dawley (SD) rats | Higher penetration rates than neutral or negative nanoparticles | 10.1016/j.ijpharm.2020.119553 |
|  |  | Polymer nanoparticles | Diphtheria toxoid | Amyloid β (Aβ) rats | Reducing AD memory impairment | 10.1007/s43440-019-00017-w |
|  |  | Thiolated chitosan nanoparticles | Galanthamine | Swiss male albino mouse | Significantly improving acetylcholinesterase levels in mouse brain | 10.1007/s13311-018-00694-0 |
|  |  | Chitosan nanoparticles | Galanthamine hydrobromide | Male Wistar rats | Reducing AChE levels and activity and the hyperphosphorylation of interleukin and tau in the brain | 10.3109/10717544.2016.1153748 |
|  |  | Polymeric nanoparticles and solid lipid nanoparticles | Tarenflurbil | Male SD rats | Nanomedicine can be transported directly to the brain via olfactory nerve pathways | 10.1016/j.ejps.2016.05.012 |
|  |  | Polyethylene glycolic liposomes | Rivastigmine | Male albino rabbits | The bioavailability of drug at plasma and brain levels increased to 4-fold | 10.1080/10717544.2017.1309476 |
|  |  | Liposomes | H102 (novel β-sheet breaker peptide) | Male SD rats | Improved spatial memory deficits in rats, increased cholinesterase levels and decreased the number and size of Aβ plaques | 10.1007/s11095-015-1744-9 |
|  |  | Nanoemulsions | Memantine | Male SD rats | Encapsulation of mevalonate in nanoemulsions maintained its antioxidant potential | 10.1080/02652048.2020.1756971 |
|  |  | Nanoemulsion of chitosan nanocapsules | P38MAPK inhibitor | Male Wistar rats | Reducing p38MAPK phosphorylation in the brain | 10.2217/nnm-2018-0496 |
|  |  | Nanoemulsion | Donepezil | Male SD rats | A new method for treating AD via nose-to-brain drug delivery | 10.1007/s13346-020-00754-z |
|  | **Microspheres** | Polymeric nasal microspheres | Hydroxypropyl-β-cyclodextrin | Male SD rats | Oxidative stress and apoptosis are inhibited in the rat hippocampus, with protective effects against β-Amyloid (1-42)-induced neurotoxicity | 10.1016/j.xphs.2016.05.017 |
|  |  | Lectin-functionalized microspheres | Rivastigmine | Male Wistar rats | Better memory retention in rats | 10.1016/j.biopha.2021.111829 |
|  |  | Microemulsion | Galanthamine hydrobromide | Male SD rats | Penetrating the BBB more easily and enhancing efficacy against oxidative stress | 10.1007/s13346-020-00739-y |
|  |  | Microemulsion | Rivastigmine tartrate | Male SD rats | Reversible increase of rivastigmine tartrate in the brain | 10.1007/s11095-017-2279-z |
|  |  | Microemulsion | Morin hydrate | Male Wistar rats | Treating memory of AD rats on day 21 | 10.1080/21691401.2016.1276919 |
|  | **Stimulus-responsive drug carriers** | Thiolated chitosan hydrogel | Donepezil | Rabbits | Increasing the mean brain content of the drug | 10.1038/s41598-019-46032-y |
|  |  | In situ gel nanostructured lipid carrier | Resveratrol | Male SD rats | Enhanced memory function and nasal mucosal permeation in rats | 10.1007/s13346-018-0540-6 |
|  |  | Trimethyl chitosan hydrogel nanoparticles | Progesterone | Male SD rats | A 5-fold increase in brain progesterone concentration after 30 minutes of inhalation of hydrogel nanoparticles | 10.3390/pharmaceutics11120657 |
|  | **Targeted and functionalized nanocarriers** | Wheat germ agglutinin nanoparticles | miRNA 132 | APP/PS1 double transgenic mice | Reducing Aβ protein and improving learning and memory functions | 10.3389/fphar.2020.01165 |
|  |  | Lactoferrin-coupled trimethylated chitosan | Huperzine A | Male Kunming (KM) mice | Higher fluorescence intensity and longer residence time in the brain | 10.2147/IJN.S151474 |
|  |  | Lactoferrin-modified nanoemulsions | Huperzine A | Male Wistar rats | Higher brain targeting than unmodified nanoemulsions | 10.2147/IJN.S214657 |
| **PD** | **Mucoadhesion** | Thiolated Polymer (nasal dry powders) | - | RPMI 2650 cells | Enhancing mucoadhesion when used *in vitro* | 10.1016/j.ijpharm.2022.122188 |
|  |  | Chitosan nanoparticles | Pramipexole | Male SD rats | Better local neurological function, enhanced antioxidant status, and increased dopamine levels in the brain | 10.1016/j.ijbiomac.2017.12.056 |
|  |  | Chitosan nanoparticles | Rasagiline | Male Swiss albino mice | Enhanced bioavailability in the brain | 10.3109/10717544.2014.907372 |
|  |  | Nanoparticle | P substance | 6-hydroxydopamine (6-OHDA)-induced PD rats | 6-OHDA-induced apoptosis was inhibited in PD rats | 10.2147/DDDT.S77237 |
|  |  | Trimethyl chitosan-modified nanoemulsion | Ropinirole-dextran sulfate | Female Swiss albino mice | High brain targeting efficiency via olfactory pathway | 10.1016/j.ijbiomac.2018.09.032 |
|  |  | Solid lipid nanoparticles | Geraniol/ursodeoxycholic acid | Rats | Facilitating the entry of geraniol and ursodeoxycholic acid into the brain at low doses | 10.1016/j.jconrel.2020.02.033 |
|  | **Targeted and functionalized nanocarriers** | PLGA combined with lectin | Levodopa | Male CD57/BL6 mice | Enhancing nasal absorption of nanoparticles with better targeting ability and lower cytotoxicity | 10.1080/10837450.2020.1740257 |
|  |  | Borneol and lactoferrin co-modified nanoparticles | Dopamine | Male SD rats | Promoting cellular uptake of nanoparticles and higher brain drug concentrations within 12 hours | 10.1080/10717544.2019.1636420 |
|  |  | Lactoferrin-modified PEG-PLGA nanoparticles | Rotigotine | Mice | High drug accumulation in the striatum after intranasal administration | 10.2147/IJN.S120939 |
|  |  | Angiopep-2 and polysorbate 80-modified liposomes | Cyclovirobuxine D | Male SD rats | Easily crossing the BBB with high drug concentrations in the brain | 10.1166/jbn.2018.2581 |
| **HD** | **Mucoadhesion** | Liposomes | Exogenous brain cholesterol | Wild-type (WT) mice | Effectively delivering cholesterol to the brain via olfactory and trigeminal nerve pathways | 10.1021/acschemneuro.9b00581 |
|  |  | Solid lipid nanoparticles | Rosmarinic acid | Male Wistar rats | Reduced deficits in walking ability, locomotion, and motor coordination transitions in rats, oxidative stress in the striatum, and unnecessary drug metabolism in other parts of the body | 10.3109/10717544.2014.880860 |
|  |  | Chitosan nanoparticles | siRNA for HTT | YAC128 transgenic mice | Reducing the expression of the erroneous gene HTT mRNA by more than 50% | 10.1016/j.jddst.2021.102517 |
