# Supplementary figures and images for: Transnasal-brain delivery of nanomedicines for neurodegenerative diseases
Source: Front Drug Deliv. 2023 Aug 11;3:1247162. doi: 10.3389/fddev.2023.1247162 (PMC12363324; doi:10.3389/fddev.2023.1247162)

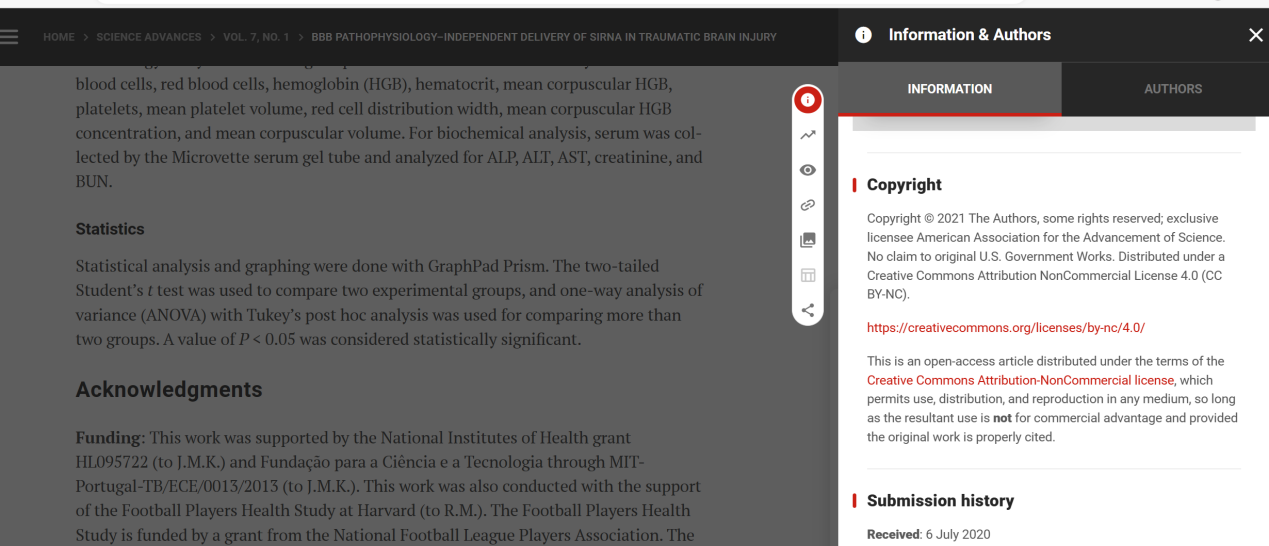


Figure 2


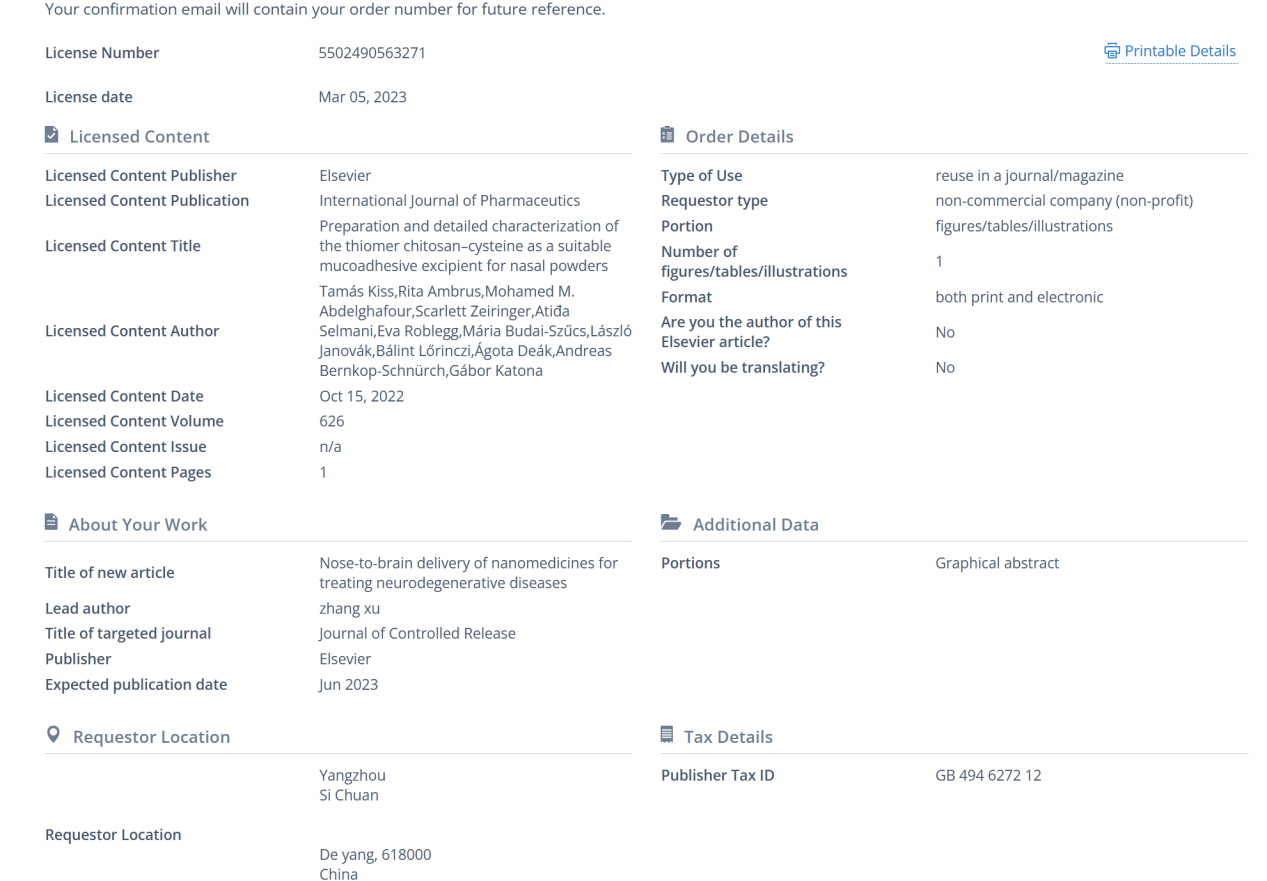


Figure 4


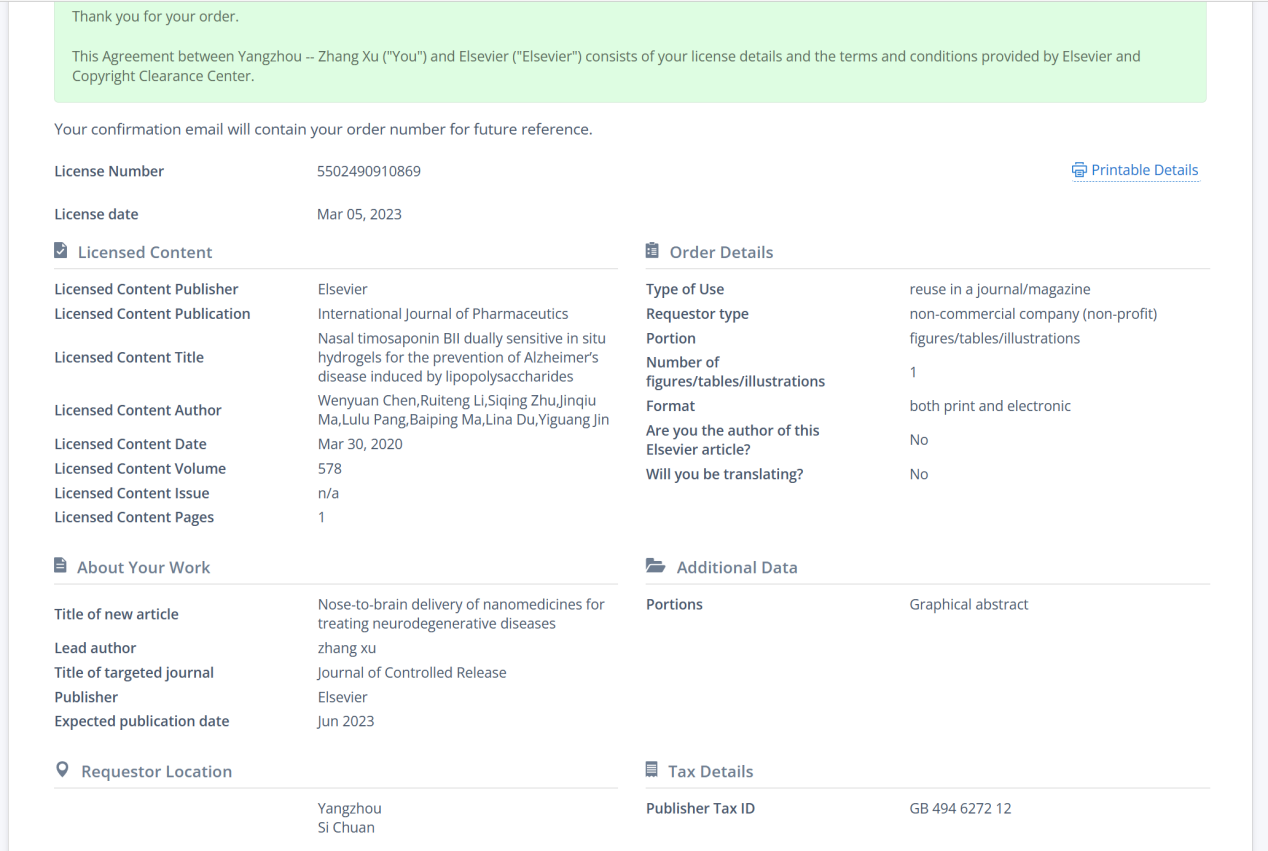


Figure 5


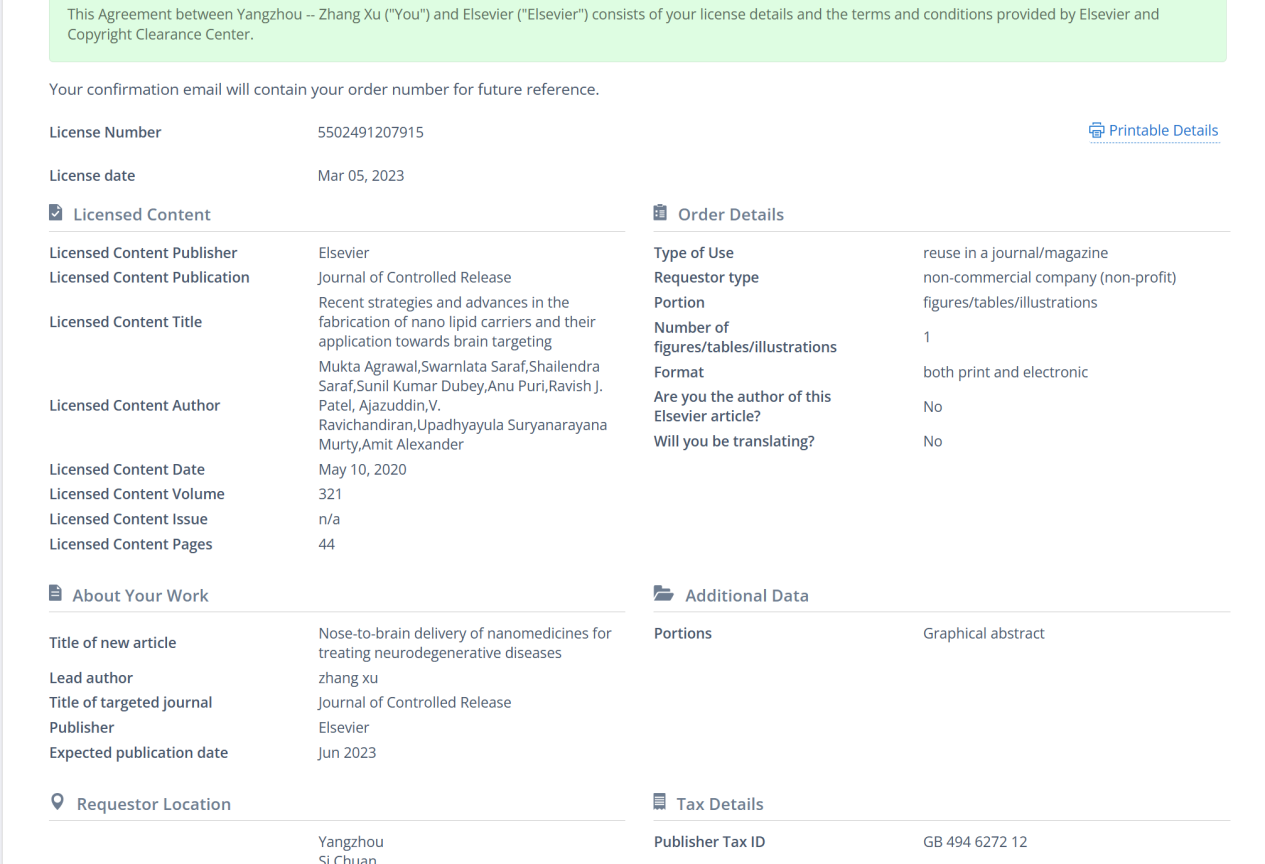


Figure 6


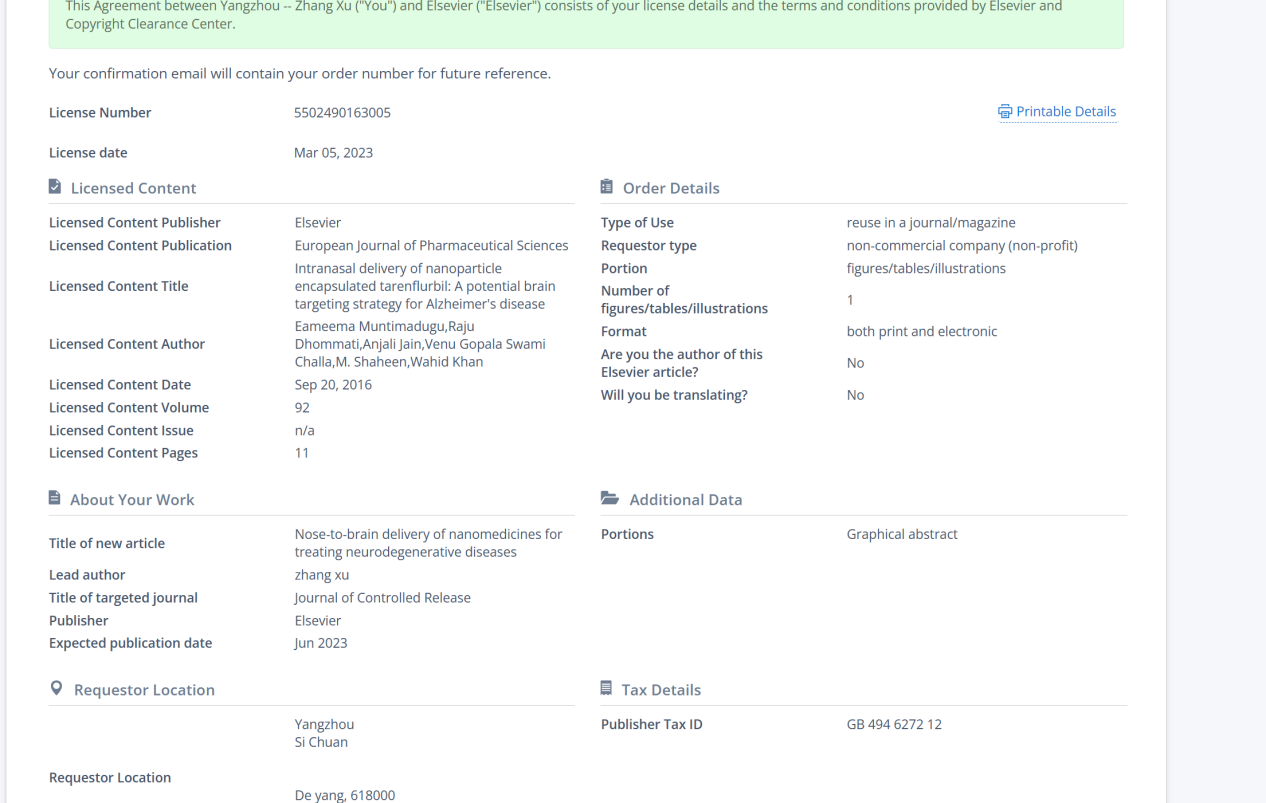


Figure 7


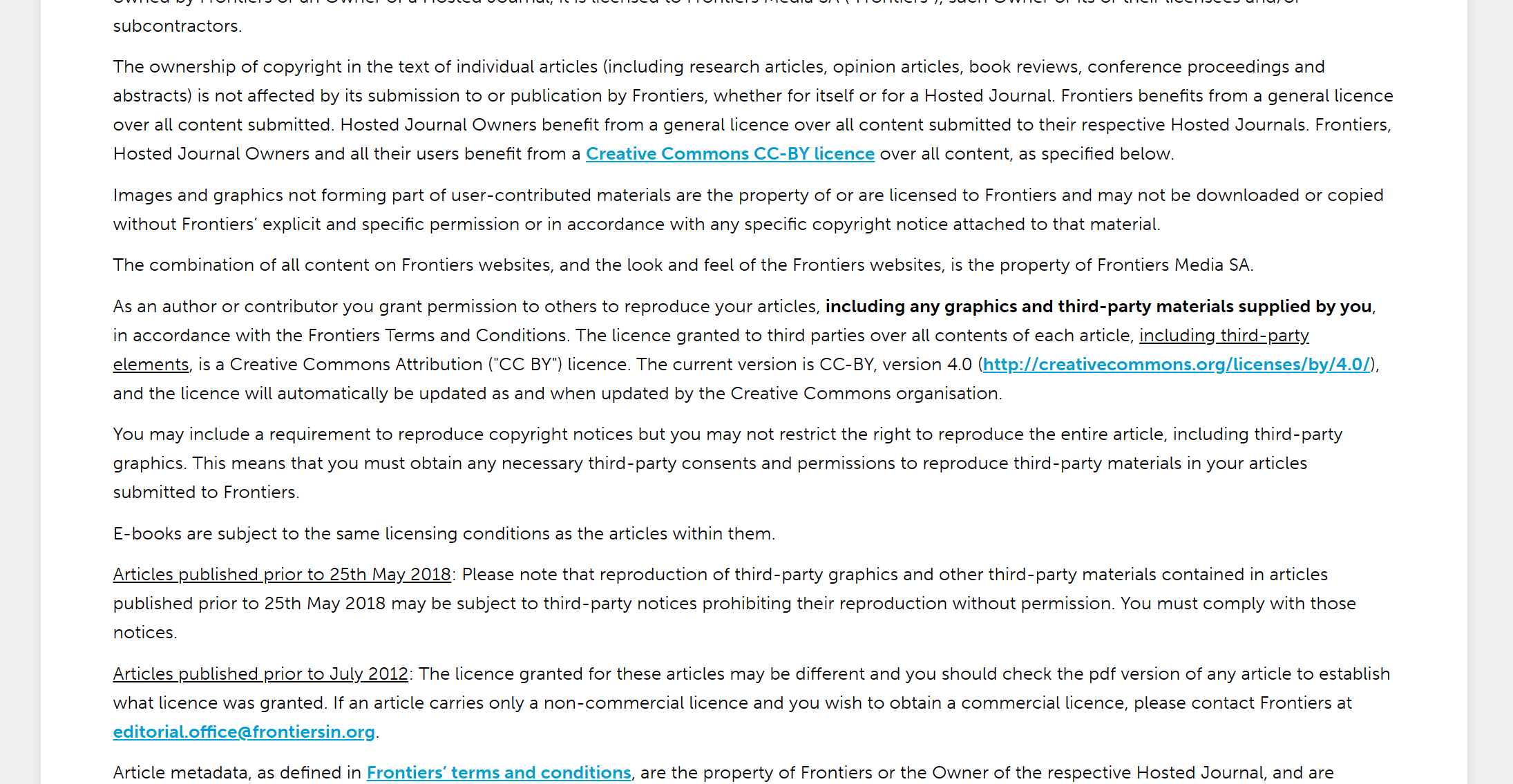


Figure 8


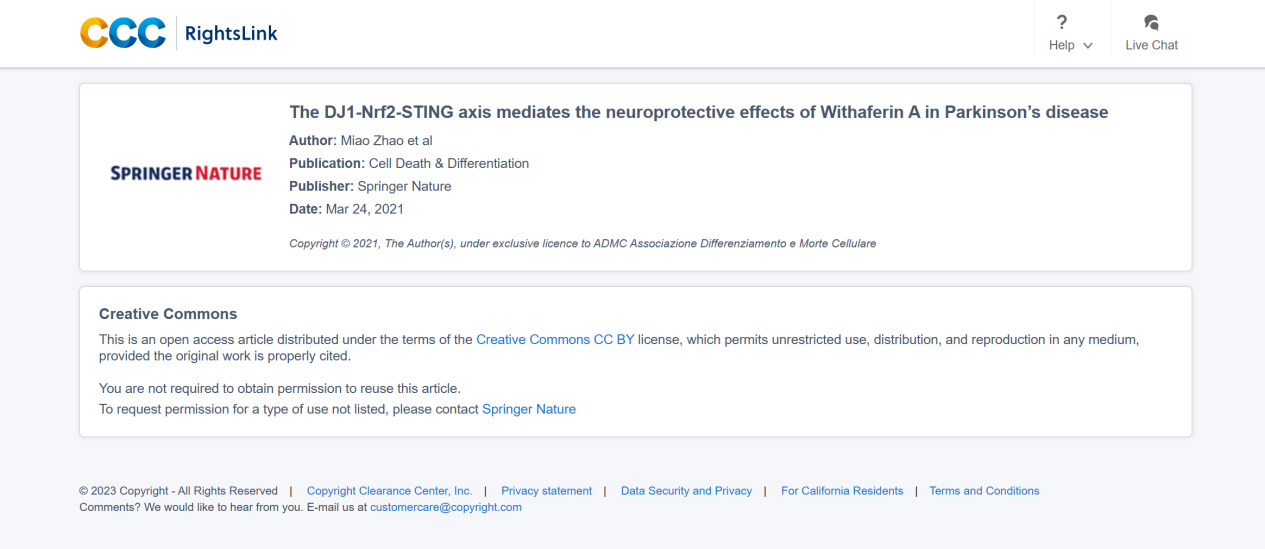


Figure 9


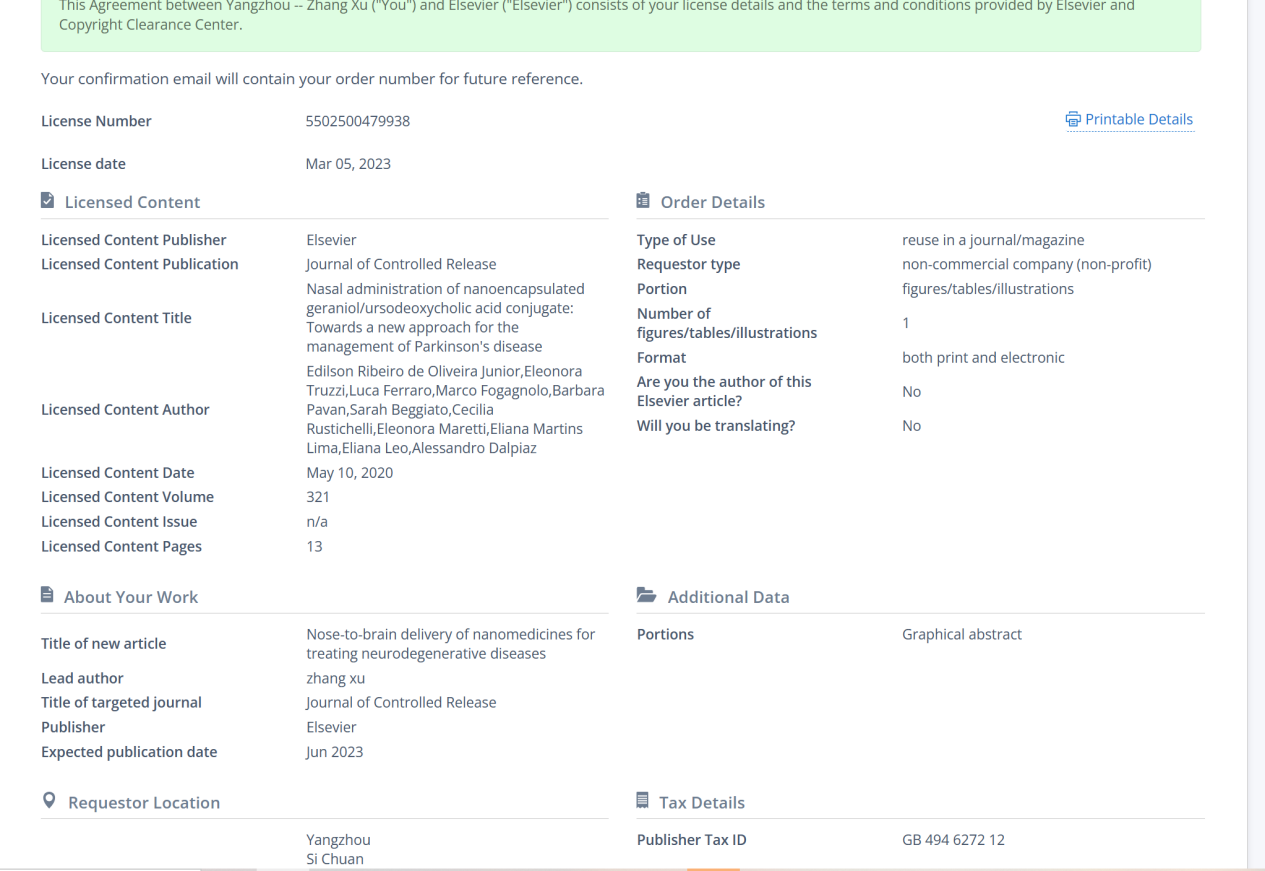


Figure 10


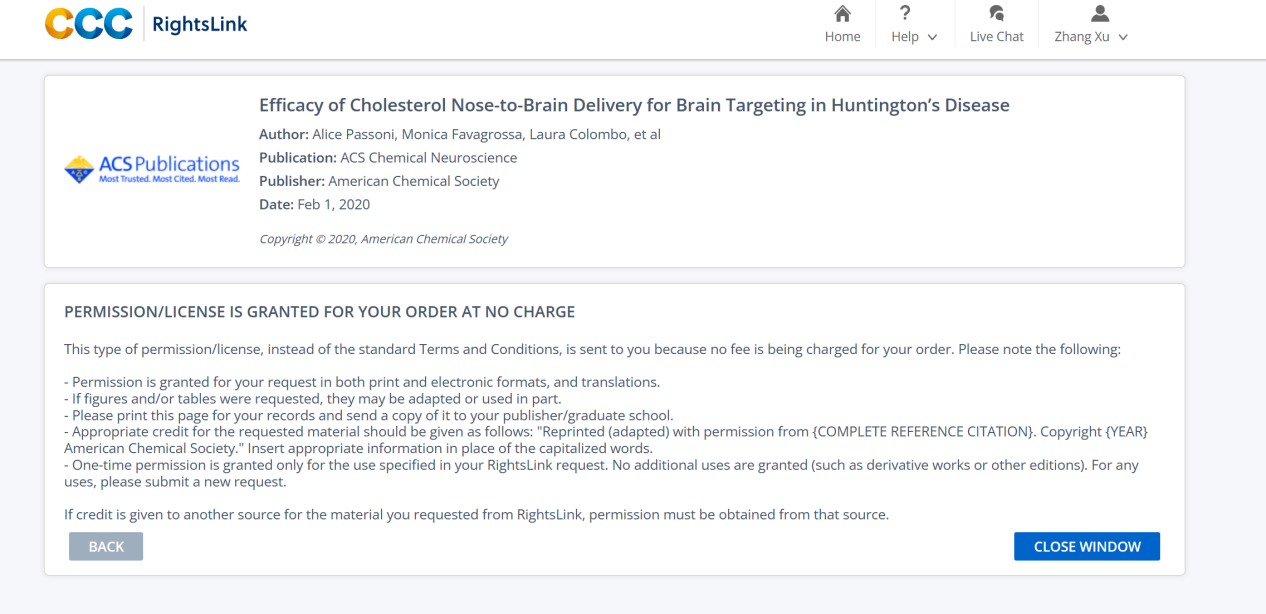


Figure 11

Supplement: Supplementary file 3 [file DataSheet1.DOCX]
